# The Open Force Field Initiative: Open Software and Open Science for Molecular Modeling

**DOI:** 10.1021/acs.jpcb.4c01558

**Published:** 2024-07-11

**Authors:** Lily Wang, Pavan Kumar Behara, Matthew W. Thompson, Trevor Gokey, Yuanqing Wang, Jeffrey R. Wagner, Daniel J. Cole, Michael K. Gilson, Michael R. Shirts, David L. Mobley

**Affiliations:** † Open Force Field, Open Molecular Software Foundation, Davis, California 95616, United States; ‡ Center for Neurotherapeutics, University of California, Irvine, California 92697, United States; ¶ Department of Chemistry, University of California, Irvine, California 92697, United States; § Simons Center for Computational Physical Chemistry and Center for Data Science, New York, New York 10004, United States; ∥ School of Natural and Environmental Sciences, Newcastle University, Newcastle upon Tyne NE1 7RU, United Kingdom; ⊥ Skaggs School of Pharmacy and Pharmaceutical Sciences, The University of California at San Diego, La Jolla, California 92093, United States; # Department of Chemical and Biological Engineering, University of Colorado Boulder, Boulder, Colorado 80305, United States; @ Department of Pharmaceutical Sciences, University of California, Irvine, California 92697, United States

## Abstract

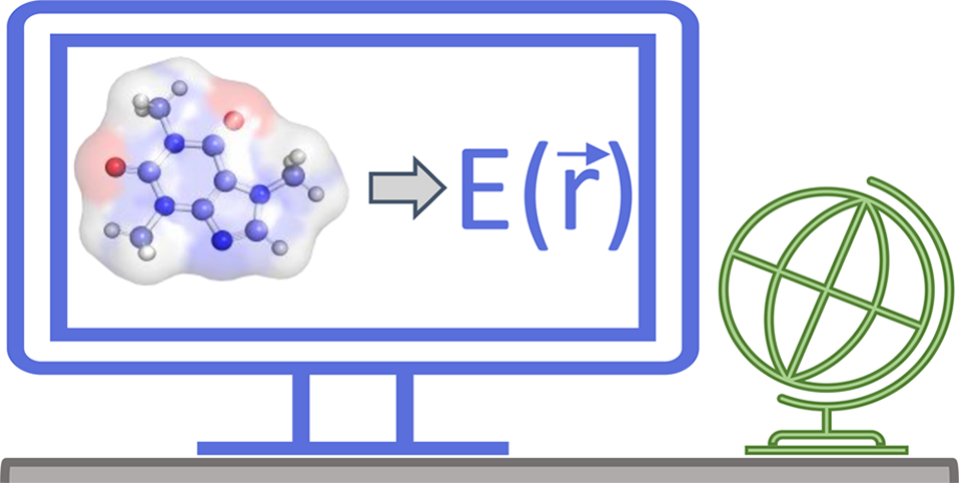

Force fields are a key component of physics-based molecular modeling, describing the energies and forces in a molecular system as a function of the positions of the atoms and molecules involved. Here, we provide a review and scientific status report on the work of the Open Force Field (OpenFF) Initiative, which focuses on the science, infrastructure and data required to build the next generation of biomolecular force fields. We introduce the OpenFF Initiative and the related OpenFF Consortium, describe its approach to force field development and software, and discuss accomplishments to date as well as future plans. OpenFF releases both software and data under open and permissive licensing agreements to enable rapid application, validation, extension, and modification of its force fields and software tools. We discuss lessons learned to date in this new approach to force field development. We also highlight ways that other force field researchers can get involved, as well as some recent successes of outside researchers taking advantage of OpenFF tools and data.

## 1 Introduction

Force fields provide a key ingredient for much of modern classical molecular modeling, providing the energies and forces in a molecular system as a function of the positions of the atoms and molecules involved. Such force fields use classical approximations to the underlying quantum chemical potential energy surface, resulting in a much simpler, if much more approximate, function of only the atomic positions. Force fields (FFs) for biomolecular and soft matter systems are typically split into valence terms (those involving atoms connected by only a few terms), which are typically fit directly to quantum mechanical (QM) data, and nonbonded terms, which use classical limits of QM derived forces, such as the 1/*r* dependence of Coulomb’s law and the 1/*r*^6^ of London dispersion forces. Most commonly in biomolecular and soft matter FFs, these nonbonded terms are two-body additive, which are relatively quite cheap, but may also frequently involve more complex (and expensive) multibody interactions such as terms involving electronic polarization.^1−3^

As a way to approximate the behavior of molecular systems relatively cheaply, force fields play a key role in biomolecular modeling and simulation, chemistry, and even materials applications.^1−9^ In drug discovery settings, they are often used to explore and guide molecular design, allowing ideas to be tested or explored in advance of their experimental synthesis and testing. Force fields thus have broad applications at diverse scales, ranging from assisting with conformer generation and estimation of geometries and energetics for small molecules^10,11^ up to helping to predict the interactions between proteins and small molecule drugs or signaling molecules,^12−21^ to protein design,^22−25^ polymer modeling,^26−28^ design of materials for separations,^29,30^ and many other areas.

Because so many different modeling techniques rely on force fields in one form or another, researchers have focused considerable development effort on building general and transferable force fields which can handle large swaths of chemistry simulated together in complex mixtures, and such force fields are now widely used across research fields. However, one major challenge in force field development is the amount of human time and expertise involved in traditional force field development. Creating a new general force field from scratch, covering most or all of normal organic chemistry and biomolecules typically takes many human-years, based on historical precedent.^1^ Thus, while there have been numerous adjustments to biomolecular force fields over the years, especially terms relating to proteins and nucleic acids,^15,31−34^ up until recently,^35−39^ the core of most of our present-day force fields, at least aside from the torsions and charges, typically date to the 1980s and early 1990s. While age in itself is not a major argument for updates, in the intervening years, computer power has grown tremendously (along with our ability to accurately compute precise estimates of varied physical properties) as well as the availability of diverse experimental and molecular data sets. Thus, force field efforts today could take a more systematic approach to fitting, taking advantage of broader and more diverse data sets and experimenting with different physical properties than those employed in these foundational efforts.

Small molecule force fields have received much less attention than biomolecular force fields, to a great extent because the chemical diversity required is far greater and the number of studies requiring any given small molecule is much smaller. Small molecule force fields have typically been developed at least in part by generalizing biomolecular force fields to cover more chemical space.^40−42^ Thus, development of small molecule force fields has lagged behind that of protein force fields, partly because of the additional chemical complexity involved and corresponding additional human time required.

Building a new *general* force field from scratch using traditional approaches would simply require too much effort over too long a time for individual academic groups to tackle the problem, not to mention the fact that typically, funding is difficult to impossible to obtain for such an academic effort.

The amount of effort involved in force field parametrization and optimization makes it surprisingly difficult to answer even basic questions in force field science. For example, the popular AMOEBA force field differs from conventional fixed-charge force fields by adding atomic polarizability, fixed multipoles, an alternate functional form for nonpolar interactions (a buffered 14–7 potential rather than Lennard-Jones 12–6) and adopting different training data and a different parametrization philosophy.^1^ If fitting were a less arduous task involving less human effort, it might be possible to determine how much each different choice impacts force field accuracy and transferability, e.g. if it were possible to fit a force field in the same manner as AMOEBA or GAFF2, while holding training data generally fixed, we could explore the impact of a single choice–perhaps taking a conventional LJ 12–6 fixed-charge force field and adding only polarization, then comparing head-to-head to determine the impact of that specific choice on accuracy, transferability, and computational cost. Such force field *science* is, to our knowledge, nearly unexplored. These issues also help motivate our desire to build new general-purpose force fields, as early efforts^43−49^ often made one specific set of choices in terms of the types of data used for fitting and the molecules considered (as well as functional form), without systematically exploring how the choice of fitting data, the composition of the training set and other factors impacted force field accuracy and transferability. A modern, more automated effort taking advantage of advances in simulation methodologies and computer power could systematically explore these issues and provide a great deal of insight into force field science.

### 1.1 What Is the Open Force Field Initiative?

The Open Force Field (OpenFF) Initiative is an open source, open data, and open science effort focused on improving force fields and the related infrastructure, such as by compiling training data and automating fitting infrastructure, thereby accelerating force field science. Our interest in this initiative was largely driven by our own frustration with the state of force field science: both the difficulty of addressing the remaining fundamental science questions in the area, as well as the unclear path toward iterative improvement of existing public force fields. Our own research fairly frequently led us to encounter force field problems when modeling small organic molecules, and in some cases even suggest solutions^50,51^ but the path toward including these in any specific force field, or getting them addressed by any force field development process, was unclear. While commercial vendors like Schrödinger have invested considerable effort in proprietary force fields, these FFs are prohibitively expensive for many to use and progress on any particular problem is dictated by a single commercial entity.

To some extent, the lack of production-level open source modern small molecule force fields springs from poor alignment of incentives. To be specific, by “production-level”, we mean force fields and infrastructure that can be scaled over large and diverse sets of input data (molecules) repeatedly in an automated or semiautomated way, without significant human attention applied to the process). Incremental improvement of existing work tends to be a poor fit with traditional academic funding mechanisms; for example, most major grant mechanisms from the US National Science Foundation (NSF) and National Institutes of Health (NIH) have “innovation” as a key review criterion. It can be hard to argue that a force field effort is innovative if what is most needed is to extend an existing effort by adding more data or exploring changes to the fitting process, functional form, or fitting data.

Yet this type of incremental innovation may be precisely what is needed to advance force field science and address lingering accuracy challenges and systematic errors in molecular modeling. Force fields and molecular modeling software already have considerable value for early stage drug discovery^1,2,5,9^ yet still have key areas where steady, often incremental, improvement is needed to improve outcomes.

At the same time, the problem of improving force fields is not necessarily an ideal fit for the pharmaceutical industry, either. Industry typically focuses more directly on human health and investments which can be expected to have a direct impact on pharmaceutical discovery and development. While individual companies invest in basic science relating to molecular modeling to varying degrees and at varying times, such investments can rise and fall with the fortunes of individual companies and changes in their management. Additionally, many companies (probably correctly) view force field and molecular modeling tools as precompetitive infrastructure—something that might be worth investing in to the extent that it improves day-to-day operations but not part of their business model. They might therefore be open to contributing some amount of money for collaborative efforts to improve force fields, but complex legal structures often preclude collaborative arrangements directly between companies in the drug discovery space.

Drawn by the vital need for high quality force fields which can be used easily at scale, in the mid-2010s, many companies began to invest in, or explore investing in, their own infrastructure in this area. However, the desire to avoid duplicate efforts and improve quality led to interest in a consortium model for force fields. In such a consortium, interested parties could pool funds and combine efforts to explore and improve force fields, while ensuring the data, results, software, and products are made available under a permissive license for subsequent reuse and further research. Ideally, the group thought, a consortium model could democratize force field development, ensuring that anyone would have the ability to make any necessary improvements to force fields in the future. The same model could allow outsiders to take advantage of the same infrastructure, data, and approaches as formal partners, accelerating innovation and facilitating crowdsourcing approaches rather than requiring success to be achieved only by a designated few.

This discussion ultimately led to the formation of the Open Force Field Consortium, a specific industry-funded consortium focused on open source/open data approaches to force fields for molecular modeling in the pharmaceutical discovery space. The Open Force Field Consortium now forms a small part of the broader Open Force Field Initiative, with the Initiative encompassing all related science and infrastructure, regardless of funding mechanism, and the Consortium being the industry-funded portion operating under one specific consortium agreement with one specific governance structure. The broader Initiative includes essentially any interested parties, including work from the Consortium’s main PIs, plus work by anyone who gets involved, such as Daniel Cole at Newcastle University (investigating new functional forms and automating torsional scans),^36,52^ and Brian Space at U. North Carolina^53,54^ (applying OpenFF infrastructure for organic solids). These collaborations generally have evolved not through a formal process but organically, with investigators reaching out to propose ideas for which working together would be beneficial for both OpenFF and these investigators.

Initially, the Open Force Field Consortium operated under the fiscal sponsorship of the NSF-funded MolSSI (the Molecular Sciences Software Institute), but eventually a new nonprofit, the Open Molecular Software Foundation (OMSF), was created to house this and other projects in the open source space relating to molecular modeling. To a large extent, the creation of OMSF was motivated by the incentive problems discussed above, with critical work in this space not being a good match in either academia or industry, as well as by the difficulty of transferring smaller amounts of money from industry to academic groups. OMSF has since expanded to host other similar software efforts, such as the Open Free Energy Consortium, the OpenFold Consortium, and Open Rosetta.

### 1.2 The OpenFF Approach Changes How Parameters Are Assigned

One way in which OpenFF seeks to reduce the human expertise involved in force field fitting is by changing how parameters are assigned. Specifically, while a reasonable amount of effort has gone into improving the automated fitting of parameters for a particular force field given input data, such as via ForceBalance,^55−57^ this approach still requires a great deal of human expertise deciding which parameters need to be fitted when building general purpose force fields. Notably, a human expert must decide how many atom types (and thus how many bond, angle, torsional, Lennard-Jones and charge parameters) are needed to represent all of the relevant chemistry, and then, given these choices and others, automated machinery can improve the values of the parameters associated with these force field terms. Atom typing rapidly becomes extremely complex even for relatively simple molecules, however.

OpenFF is not alone in the space of avoiding traditional atom typing, nor the only new force field effort. The Automated Topology Builder framework also takes an alternate approach to typing and parameter assignment^58^ as does TAFFI,^59^ and the XtalPi/Pfizer XFF force field uses a somewhat similar framework to advance an alternate force field effort.^38^ Other machine learning frameworks like Espaloma,^60,61^ Grappa,^62^ MACE,^63^ DMFF^64^ or other differentiable frameworks^65^ provide an interesting alternative and potentially promising future direction, as well.

#### 1.2.1 OpenFF Seeks to Assign Parameters via Direct Chemical Perception Rather than Indirect Chemical Perception

To automate the entire force field generation process, or at least make this possible, OpenFF sought to reduce the human expertise required even in early stages, such as atom typing. Specifically, our goal was to eliminate predefined atom types and instead move to a chemical perception language which can be adjusted as part of a force field development process, paving the way for further work to automate even the typing portion of force field generation. In particular, here, we implement this by encoding force field parameters using the SMARTS substructure search language.

**Figure 1:**
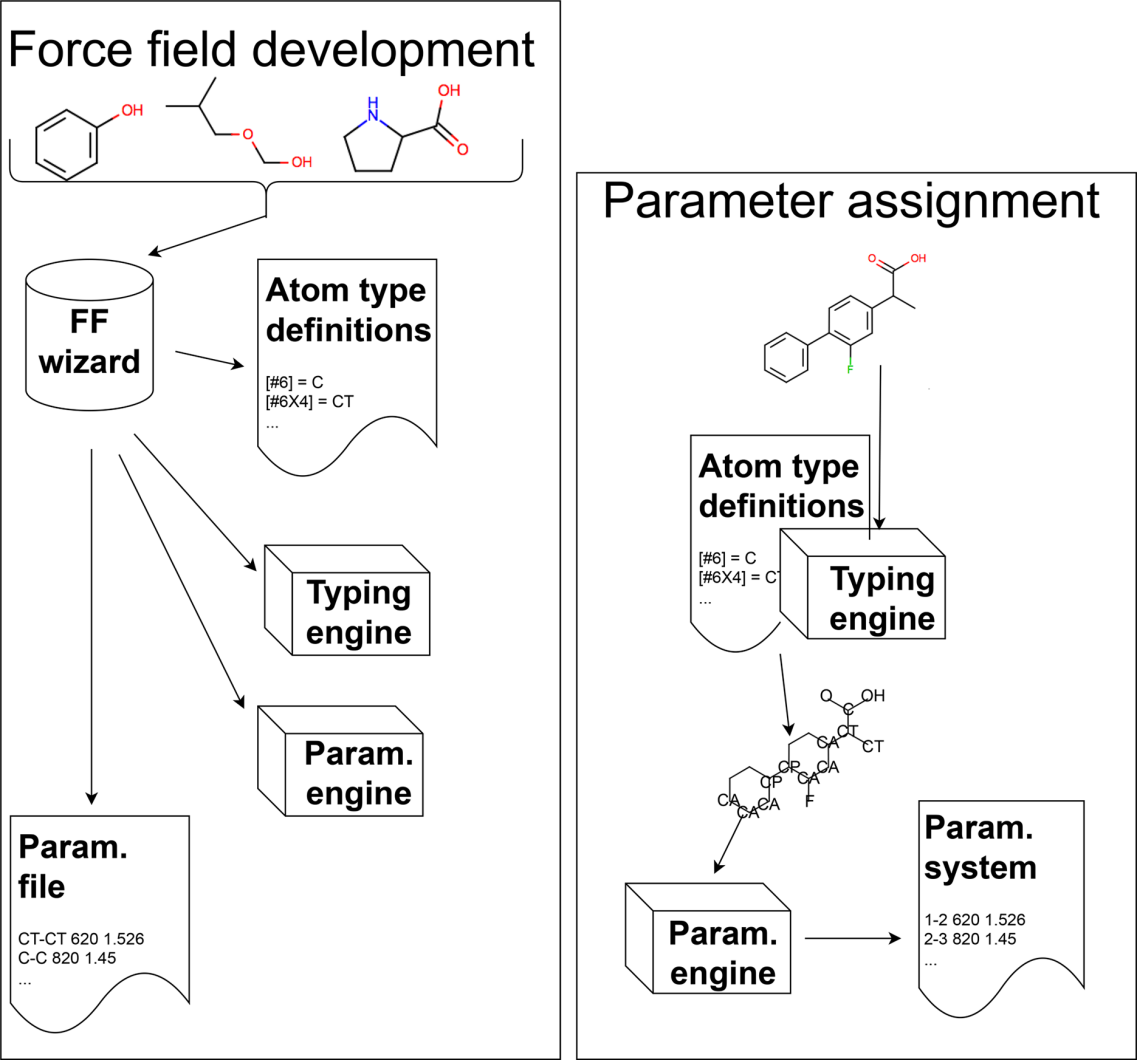
Indirect chemical perception requires that a library of atom types encodes all potentially relevant chemical environments. Force field assignment via indirect chemical perception requires several stages of processing. First, in the force field development process (left) a human expert (“wizard”) considers a set of molecules which the force field should cover and decides which chemical environments will be important to treat separately, choosing a set of atom types to bin this chemistry and tabulating or encoding these atom type definitions. The expert then encodes a typing engine which can assign these atom types to arbitrary molecules, writing out a chemical graph with atoms (nodes) labeled by atom types. Once this engine is in place, the expert separately encodes a parametrization machinery which will read in labeled chemical graphs and assign force field parameters based on atom types, often from a lookup table called a parameter file. This engine will write out the result to a file containing a parametrized system suitable for simulations. The expert also develops the parameter file which will be used by the parametrization engine. Second, in the parameter assignment process (right), a specific molecule or system is input into the typing engine previously developed, which applies the atom type definitions and writes out a labeled chemical graph. This labeled graph is then processed by the parametrization engine to produce a parametrized system suitable for simulation. This process is indirect—the parametrization engine considers a labeled graph, not the molecule itself. Thus, in this final step, all of the relevant information about distinct chemical environments *must* be encoded by the atom types and other information in the graph (in AMBER-family force fields, just the atom types and connectivity). Figure adapted from ref (67). Available under a CC-BY 4.0 license. Copyright 2018, Mobley et al.

Atom typing can be thought of as a type of *indirect chemical perception* (Figure 1), where a molecule or molecules are processed via some machinery to assign labels to atoms (atom types) and then these labels are subsequently processed to assign parameters. Thus, the key to success is ensuring that the atom types encode all of the relevant information but no *unnecessary* information, as once parametrization is begun, the atom typing rules are considered fixed. Subsequent addition of new atom types—for example, to extend the force field into new areas of chemical space—creates enormous difficulties in how existing parameters should be adjusted to accommodate the need to fit newly created parameters.^8^ We note that hierarchical schemes can assist with this, e.g.,^66^ and thus a hierarchical scheme provides part of our solution as well.

Force field parameters could instead be assigned by a process of *direct chemical perception*, which might bypass atom typing altogether and would assign parameters to individual atoms, bonds, angles, or torsions by processing the full molecular graph *directly* via a chemically aware engine. To see the distinction, note that force fields in the AMBER force field family do not retain bond order when assigning parameters, so if any bond order information is necessary, this must be encoded in the labels or atom types themselves, as we discuss further below. In contrast, a tool doing direct chemical perception could use information about a molecule such as bond order, as it operates directly on the molecular graph itself rather than an intermediate labeled graph that no longer retains bond orders or access to other molecular properties. Atom typing can therefore be thought of as a lossy compression of the local chemical structure, providing only the information about the chemical environment that is preserved in the atom type definitions.

In our view, while atom typing has been extremely helpful in developing general and relatively transferable force fields that have allowed a great deal of progress in applications of molecular modeling (MMFF94,^68,69^ GAFF,^42^ and CGenFF^70^ have been crucial in enabling widespread modeling of protein–ligand interactions), we believe it has also impaired force field science and force field development and we hope to change that. Atom typing requires a human expert, and poses an arduous task introducing “a certain degree of ambiguity and arbitrariness”.^71^ Given the expertise required, then, most work on general purpose (bio)molecular force fields is done by select individuals in just a handful of groups. The chemical perception for atom typing is typically hard-coded into software tools where it is often both invisible and hard to modify (although there are efforts to change this^72^). Overall, this impairs force field science because very few individuals or groups have the necessary expertise to modify, extend, or even troubleshoot the available expert systems for atom typing, although some efforts are being made to improve this, such as by hierarchical atom typing.^66^ Atom typing also provides a key place where early decisions or even mistakes can lead to subsequent problems for force field development which are hard to overcome. For one, atom typing makes an up-front decision as to how to bin chemical space. Once separate atom types are assigned, it is difficult to bin chemical space differently, even if the data might warrant it. Additionally, an introduction of a new atom type to fix a problem with one valence term results in a proliferation of parameters for all valence terms. To apply automated parametrization machinery when many equal parameters exist (such as the 16 sets of Lennard-Jones parameters for carbon in GAFF/GAFF2 which only have three distinct values^40^), a human expert would have to designate which parameters should be constrained to be identical versus which should be allowed to be distinct.

The complexity of atom typing and the lack of independence of the chemical perception for different parameter types also makes parametrization vastly more complicated. For example, AMBER16s GAFF 1.8 has 6,387 lines of parameters and GAFF2 (version 2.1) has 6,796. A key question for applying automated methods like ForceBalance is how many of these constitute parameters which should be fitted separately, versus how many should in fact be equivalent, such as the multiple identical Lennard-Jones parameters for carbons in biphenyl, but are different only because of indirect chemical perception. One concrete example of this issue is the CA-CA carbon aromatic bond in AMBERs parm96^45,73^ and parm99 sets,^74^ which has a length of 1.40 Å. The CA-CB bond, which is defined between substantially the same types except that CB is an an aromatic carbon at the junction of 5- and 6-membered rings, such as in adenosine and tryptophan, has a length of 1.404 Å. The difference between these two bonds is very small at only 0.004 Å, with identical force constants, and with no clear data indicating that this difference is warranted or truly significant,^73^ given the precision of the calculations.

**Figure 2:**
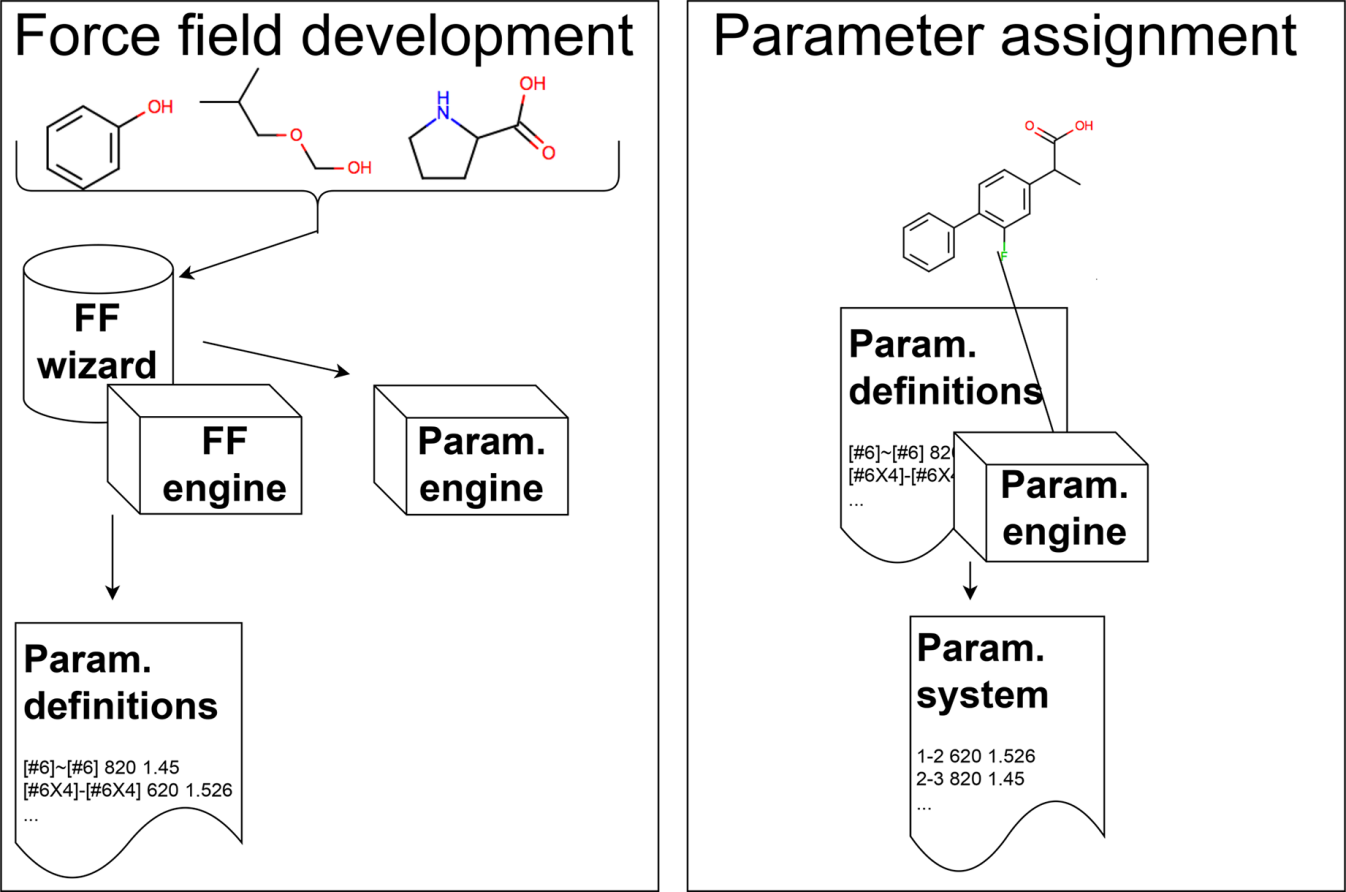
Direct chemical perception eliminates the need to encode all relevant chemical environment information in arbitrary predefined atom types. Force field assignment via direct chemical perception works on the full chemical graph of the molecules involved (including elements, connectivity, bond order, etc.), rather than first encoding information about the chemical environment into a complex set of predetermined atom types. First, in the force field development process (left) a human expert (“wizard”) and/or an automated method (a force field engine, FF engine) considers a set of molecules which the force field should cover (as well as potentially input data) and develops a force field to cover this chemistry, producing a set of parameter definitions and a parametrization engine that can apply these to molecules. Second, in the parameter assignment process (right), a specific molecule or system is input into the parametrization engine previously developed, which processes the molecule and uses the parameter definitions to apply force field parameters, producing a parametrized system suitable for simulation. The parameter assignment process is direct; the parametrization engine acts directly on the chemical graph of the molecules comprising the system, so all chemical environment information provided (or computable) is available to the engine. Unlike indirect chemical perception, there is no intermediate step of assigning atom type labels to a molecular graph; parameters are assigned directly based on the chemistry. Figure adapted from ref (67). Available under a CC-BY 4.0 license. Copyright 2018, Mobley et al.

Direct chemical perception (DCP) allows direct assignment of parameters via processing the full chemical graph of molecules, avoiding the limitations of atom types (Figure 2)—instead of assigning parameters by processing a connectivity graph labeled with predefined atom types, DCP can assign parameters via operations acting directly on the full chemical or molecular graph. For our purposes, the “full chemical graph” here is defined as the standard valence bonded representation of the molecule with explicit hydrogens, formal charges, and an aromaticity model applied. Essentially, DCP means using a chemical perception language to assign force field parameters based on molecular fragments. DCP can be used to encode traditional atom type-based force fields by encoding the same chemical perception, so it can even reproduce pathologies or complexities associated with atom typing (with enough effort) such as the complex treatment of bridgehead atoms in GAFFs handling of biphenyls. Direct chemical perception avoids these problems naturally. For example, the bond between aromatic rings in biphenyl is a single bond, and thus DCP can easily recognize it as requiring different bonded parameters than the aromatic bonds within the aromatic rings.^75^ Additionally, since the parametrization engine has access to the molecular graph, it has bond order information as well as full access to all information about the chemical environment. Thus, a variety of tools can be applied in parametrization, including (if needed) electronic structure calculations.

Direct chemical perception avoids hiding the chemistry addressed by individual terms of the force field under an additional layer of encoding which can obscure the intent. For example, consider parameter assignment for valence parameters within ring systems of various sizes. In todays fixed charge force fields, bond stretch parameters within such rings are dominated by the order (single, aromatic, or double) of the bonds involved, with modulation in some cases by the number of attached electron withdrawing or donating groups, but the size of the rings involved plays very little role in the bond stretch parameters. In contrast, angle bending parameters for the same rings show almost the exact opposite behavior; bond order matters comparatively little because the angle is primarily dictated by the geometry of the ring, whereas the size of the ring plays a huge role in determining the equilibrium angle. Thus, it seems that one type of chemical perception, focused primarily on bond order, is appropriate for assigning bond stretching parameters, whereas another is more appropriate for angle bending parameters.

However, indirect chemical perception typically applies the same chemical perception for all force types, so if we need to introduce new atom types to capture the correct geometry of rings, we will simultaneously be introducing new bond stretching parameters, whether we want them or not. GROMOS is a notable exception,^76,77^ using separate atom typing for valence versus van der Waals terms, though similar concerns still apply (specifically, generalization of the GROMOS force field to new molecules has often been done by hand by human experts (“assigning parameters based on analogy” as one report put it^58^), though approaches like the Automated Topology Builder aim to change this^58^). Direct chemical perception allows us to easily avoid this and focus on the (potentially unique) chemical effects which are important for individual force terms. DCP also allows the issue of generality versus accuracy to be explored specifically for individual parameters in the force field without requiring coupling among all parameters. For example, with DCP, one can easily explore whether introduction of a new Lennard-Jones parameter improves agreement with specific data, without necessarily requiring new torsions to be introduced to the force field. In the long-run, we believe DCP makes force fields more easily extensible simply because the chemical perception is not hard-coded into a piece of software by an expert.

In the OpenFF Initiative, we use a specific implementation of direct chemical perception, based on the chemical query language SMARTS^78^ and its SMIRKS extension, as the basis for our SMIRKS Native Open Force Field (SMIRNOFF) format. We use DCP to assign both bonded parameters (bonds, angles, torsions) and nonbonded parameters (vdW and electrostatics terms). Specifically, we currently assign atomic Lennard-Jones parameters using DCP, and use Lorentz–Berthelot combining rules to parametrize interatomic interactions. While directly parametrizing interactions between atoms would offer more flexibility, this would result in a huge expansion of parameters in the force field and therefore has so far remained a prospect for future exploration. Electrostatics parameters assigned using DCP are generally done so to compensate for deficiencies with our charge model, such as lack of coverage for particular ion species, or poor scaling for larger molecules such as proteins (see section 3). SMIRKS, and the SMIRNOFF format, can dramatically reduce the complexity (in terms of number of apparently independent parameters) in existing force fields while still yielding force fields of broad generality and allowing a variety of new innovations which would be quite difficult in typical force fields.

Direct chemical perception allows a dramatic simplification of force field typing and greatly reduces redundancy. For example, typical OpenFF force fields have a few hundred lines of parameters in contrast to other force fields which have thousands to hundreds of thousands of lines of parameters, yet OpenFF accuracy, depending on the measure, has been better, comparable, or at least not dramatically worse.^79^

#### 1.2.2 This Direct Chemical Perception Approach Facilitates Inferred Types

OpenFF’s direct approach to chemical perception allows typing to be manipulated as part of the force field development and assignment process. Thus, early OpenFF efforts in this area experimented with certain approaches for automatic derivation of types in a data-driven manner.^80,81^ However, the combinatorial complexity of potential types have led to later variations of this. For example, as we discuss further below, OpenFF Initiative researchers have been exploring “bespoke” parametrization of molecules, where some aspects of typing and parametrization can be refined in application to a specific molecule or a series of molecules.^52^ Others are exploring data-driven refinement of types via proposed splits and merges.^82^

### 1.3 The OpenFF Approach to Open Data, Open Software, and Open Science

A key driving philosophy of OpenFF is the concept of open science. This concept of openness applies in three main areas, here: open-source software, open data, and an open scientific discovery process. We believe open data and open software is essential to this open scientific process, furthering force field science by enabling external researchers to focus on building on our work rather reproducing it. With free access to the same tools that we use, scientists can easily expand on our work, conduct their own experiments, and compare performance.

**Open data**: We aim to ensure all data sets used in the fitting or benchmarking of released force fields abide by the principles of FAIR:^83^

- Findable and Accessible: OpenFF fits and benchmarks parameters both to quantum chemistry (QC) data sets and physical property data sets. For QC data sets, which we typically generate ourselves, we assign unique identifiers (data set names and versions), and we maintain software (OpenFF QCSubmit) to act as an interface for retrieving the data sets. Data sets are deposited in MolSSI’s QCArchive database so that they can be easily reused by researchers across the Initiative and field, regardless of where they are based. As of this writing, MolSSI’s QCArchive serves as a public archive of all generated quantum chemical data, though in the long term it is likely to shift to retaining only currently active data sets, in which case OpenFF’s archival fitting data sets will continue to be made available with force field releases and as permanent releases on Zenodo. For data sets we use from other sources, e.g. our physical property data sets, we commit to only using data with an open license. Where licensing allows, we distribute the specific subsets of data used for training and fitting our force fields. If this is not possible, we release the scripts we use for retrieval and curation. Our force fields are also named following semantic versioning for easy identification.
- Interoperable: in our force field releases, where possible, we release the actual targets used for fitting in both human- and computer-readable formats. For valence fitting targets where we own copyright, we release a) text files designed for ForceBalance, in addition to b) JSON files for the OpenFF QCSubmit library. For physical property data, where licensing allows, we release the actual targets used in human- and computer-readable CSV files and JSON files for the OpenFF Evaluator library. We furthermore release our benchmarking results as CSV files.
- Reusable: We release our data sets under a permissive open license (typically CC-BY). All QC data sets in our qca-data set-submission repository are generated and described with version identifiers and rich metadata describing the scope (size, number of conformers, elements covered, computational procedure) of each data set, as well as the motivation and the procedure for generation. All data sets include a “provenance” section that lists the versions of key packages involved. We have also recently begun including the full Python environments used to generate the data set for improved reproducibility.

**Open Software**: The OpenFF approach to software goes hand in hand with our commitment to open data. We release all software under permissive open-source licenses, typically MIT and BSD-3, to allow as many people as possible to easily use and benefit from our code. We both develop our own libraries, and contribute to other key libraries in the open-source molecular modeling space. As described below in the “infrastructure toolkit” section and “alternative functional forms” section), we provide multiple interfaces for users that allow them to extend and build on our code. We strive to have comprehensive documentation, tutorials, and examples on how to use our software and best practices. Again as with the data, we aim to follow the FAIR4RS principles.^84^

- Findable and Accessible: our libraries are hosted, and developed through GitHub. They are released and published on GitHub, Zenodo, and conda-forge, where they can be installed with standard Python package managers. Each release is uniquely labeled with a version identifier that either follows semantic or calendar versioning standards.
- Interoperable: our software is designed to be interoperable with standard formats, major cheminformatics toolkits and popular molecular modeling engines, as expanded on in the discussion of OpenFF Interchange below.
- Reusable: we use standard versioning schemes and release our packages with clear specifications on required dependencies through the software channels above.

**Open Scientific Process:** OpenFF is designed for the scientific process to be as open as possible. We post all presentations from our preprints and meetings publicly on our Web site. We encourage other investigators to use our tools and to participate in discussions. The BespokeFit package (described more below) is an example of a project that developed through contact with an outside investigator who had shared interests.

If you are interested in participating or collaborating with OpenFF, we encourage you to get involved via our organization’s GitHub repositories and discussion board, as well as our examples, workshops, and documentation. We are also happy to connect interested collaborators with team members working in relevant areas. Overall, our hope is that in the long run, the community will take advantage of OpenFF data sets and infrastructure (as well as their own contributions) to push forward force field science dramatically so the field as a whole will benefit from dramatically improved force fields, whether they come from our project or elsewhere.

A word about decision-making is warranted. To a large extent, OpenFF strives to make decisions based on running scientific or fitting experiments rather than in a philosophy-driven manner, as the latter often results in wasted time and effort. To give a concrete example, early OpenFF efforts selected one particular choice of QM basis set and level of theory for initial fitting work (based on careful examination of literature data),^85^ with the expectation that this would have to be improved at a later date (with a corresponding refit of all force fields) after fitting machinery was in place following a more systematic benchmarking effort. However, this more systematic benchmarking effort seems to suggest that our initial choice–which was relatively carefully informed based on the literature–was indeed adequate^86^ so we continue to rely on the same QM choices. We also have to make choices about weighting factors used for ForceBalance fitting,^85^ and rely heavily on testing and benchmarking of different fitting experiments to see which results in best accuracy and transferability. Recent choices to move to fitting to mixture data rather than pure solution data^39,87^ were informed by fitting experiments as well. Likewise, use of vibrational frequency data in fitting hurt force field accuracy (at least with our present data sets and machinery) so this was dropped.^39^

That said, resources may not be adequate to systematically explore all choices on the desired time scale, so the main OpenFF Consortium is led by its governing board in deciding where to invest resources. Long-term, this will likely result in a diverse ecosystem of force fields and exploration done by the broader OpenFF Initiative (see e.g. the double exponential work of Cole and collaborators^36^), with only certain innovations being picked up by the more narrowly focused Consortium.

### 1.4 Synergy with Other Open Science Efforts

OpenFF promotes open collaborations for the larger benefit of the molecular simulation community, and owes its success to all the contributions from both upstream and downstream developers in adapting our software stack and providing feedback on our science efforts. We rely on many external software packages and public databases in training and testing of our force fields. At the most basic level, simulation engines such as OpenMM^88^ and GROMACS^89^ are critical for enabling us to employ our force fields in simulations. PMX from the Gromacs community^90^ and the Open Free Energy initiative have been crucial in benchmarking our Parsley and Sage force fields with binding free energy calculations.

OpenFF is furthermore only able to obtain the data sets we use to fit and benchmark our force fields by building on top of earlier work by the scientific community. We use physical property data from publicly available databases such as ThermoML,^91^ MnSol^92,93^ and FreeSolv.^94^ For training and benchmarking valence parameters we generate substantial quantum mechanical (QM) data. This QM data generation relies on the electronic structure package Psi4 and the QC infrastructure created by MolSSI (QCFractal, QCPortal, QCEngine), along with QCArchive for storing the generated data according to FAIR data principles.^95,96^ The sustained development of Psi4, incorporating new quantum chemical methods and maintenance of the aligned QC packages to modern programming standards, has been crucial in fueling our science efforts at scale.

For example, the infrastructure made it easy to generate huge data sets such as the protein data sets we are using to train the next generation fully consistent protein and small molecule force field, Rosemary. These protein data sets include two-dimensional torsion scans of protein backbone dihedrals, which easily runs into 300 K or more constrained optimizations per data set (with around 6000 constrained optimizations per 2D torsion scan of 576 (24 × 24) grid points).^97^ Another joint effort with OpenMM was generating the SPICE data set.^98^ This data set, which contains more than a million single point energies and forces, was enabled by OpenFF’s QCSubmit and MolSSI’s QC software stack with Psi4 being the calculation engine. OpenFF’s Industry Benchmark set^79^ is another example of collaboration with like-minded people in industry who want to push for open standards in benchmarking force fields. This in turn has benefited others in the molecular modeling community; the authors of the XFF force field included the OpenFF Industry Benchmark in their validation data set.^99^

## 2 Current Progress

### 2.1 New Generations of Force Fields

Our first SMIRNOFF format force field was SMIRNOFF99Frosst.^75^ This force field was an adaptation of Bayly’s AMBER-family (or GAFF-sibling) parmFrosst force field^100^ into the direct perception SMIRNOFF format.^75^ No refit was performed at this point; it was solely a representation of this older force field in using SMIRNOFF spec described above. Although not an advance in force field quality, it was an important conceptual advance demonstrating the implementation and ease of use of direct perception concepts.

Following SMIRNOFF99Frosst, we released our first actually refit force field, OpenFF 1.0.0 “Parsley”. SMIRNOFF99Frosst was used as a starting point for fitting of Parsley, with significant optimization of the valence parameters through fits to geometries and energetics from an extensive set of QM calculations.^85^ Since both GAFF and Parsley share roots in the AMBER family of force fields, this meant that their nonbonded parameters were virtually identical.^85,101^ The fixed charges used the AM1BCC-ELF10 charge model,^102^ with both AmberTools and OpenEye AM1BCC charging workflows being recognized as acceptable choices to generate these charges, despite the fact there can be some small differences between the two programs in the implementation.

Parsley serves as the code name for the entire OpenFF 1.x series force fields, and subsequent releases in this series included several important updates and bug fixes. Parsley 1.1.0 included the addition of additional nitrogen-centered improper torsion terms to better describe key planar and pyramidal structures that can be difficult to differentiate.^103,104^ Parsley 1.2.0^105^ included a major redesign of quantum chemical training data sets to improve diversity and coverage and better represent core chemistry, followed by a full valence parameter refit to this new data set. Updated training data^106^ resulted in significant improvement in relative conformer energies, optimized geometries, and torsional profiles with respect to accurate high-level *ab initio* data when compared to Parsley 1.0.0. Parsley 1.3.0 added new torsion parameters for dialkyl amides to improve amide torsional energy profiles^107^ and in Parsley 1.3.1 we corrected a minor regression for sulfonamides accuracy.^108^

**Figure 3:**
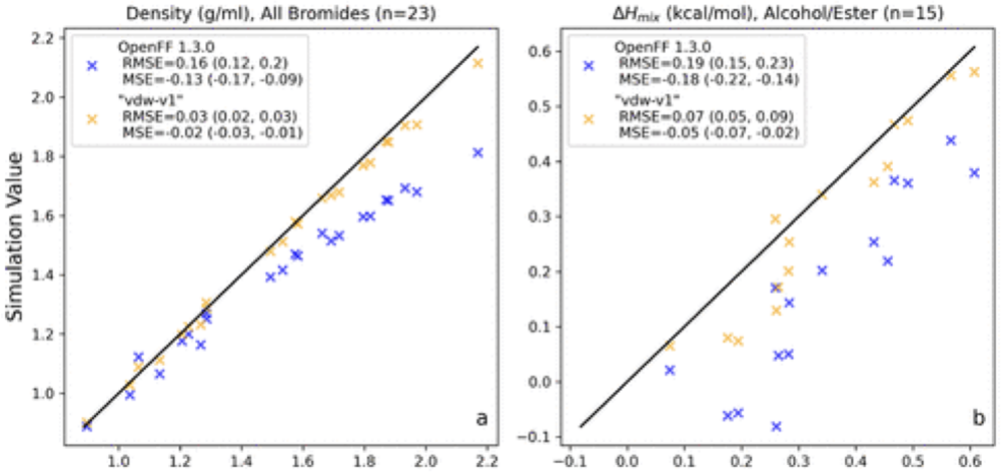
Selected categories of physical property training data, before and after LJ optimization. These plots show parity between experiment and simulation for physical properties in the training set, before (Parsley 1.3.0), and after LJ training. “MSE” in the panel legends refers to the mean signed error (bias) of the data set. Panel a shows correction of systematic error in bromide density prediction, particularly in data-based reduction in [#35:1] *R*_*min*_/2. Panel b shows correction in *ΔH*_*mix*_ of alcohol/ester mixtures after training to mixture data. As the ester group is a hydrogen bond donor but not acceptor, optimization of energy and density of pure esters would not recognize the need to create favorable interactions with hydrogen bond donors; only by including thermodynamic properties of liquid mixtures in fitting can we properly treat complex mixtures of molecules. Figure adapted from ref (39). Copyright 2023, American Chemical Society.

The OpenFF 2.0 release was code-named “Sage” and incorporated a continued refinement of valence terms and a refit of LJ parameters.^39^ Sage included substantial new work retraining the valence parameters used in Parsley, but the largest update was retraining of select Lennard-Jones (LJ) parameters to physical properties. Previous OpenFF LJ parameters were inherited from predecessor force fields (AMBER parm99^109^ and parmFrosst^100^). In Sage, LJ parameters were optimized against condensed phase physical properties, including enthalpies of mixing and densities measured for both pure and binary mixtures. We found these mixture properties to result in better force fields than optimizing to pure properties and heats of vaporization alone (Figure 3).^110^ Fitting to mixture properties that included water used TIP3P as the water model, meaning Sage should be used with the TIP3P water model, though future work is expected to change this (see discussion about co-optimization of the water model below). We also tested Sage on cross-solvation free energies (transfer between solution environments),^39^ as some other researchers have done;^111^ such data may be interesting for fitting in the future, though it remains somewhat sparse for many regions of chemical space. Overall, Sage remains essentially an AMBER-family small molecule force field, and thus AMBER force fields for proteins and nucleic acids are tested and recommended with OpenFF small molecule force fields.^39^

**Figure 4:**
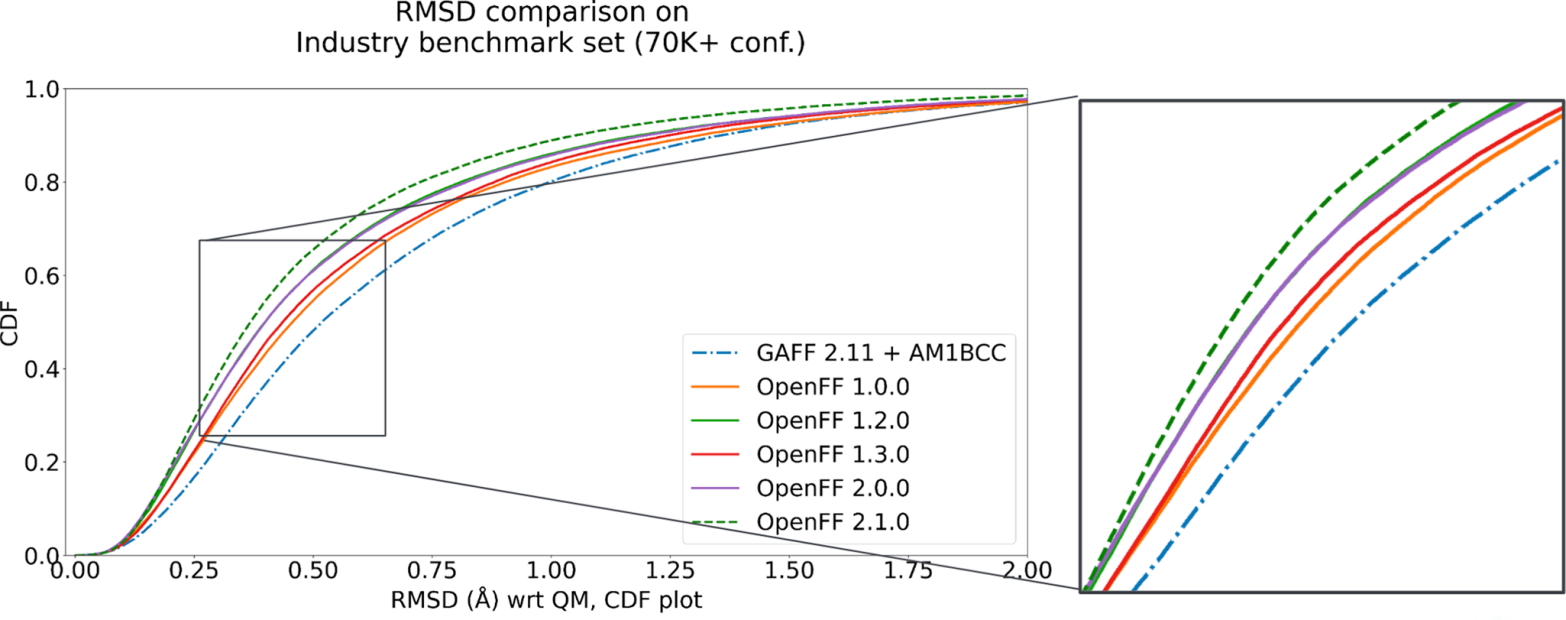
Quality of optimized geometries relative to QM reference data on our benchmark data set. Shown is a cumulative distribution function (CDF) assessing what fraction of QM optimized geometries are predicted correctly (within a given RMSD cutoff) by MM optimizations for molecules in OpenFF’s public industry benchmarking set, consisting of 9847 molecules with a total of more than 70K conformers. A higher CDF is better. The QM reference approach is B3LYP-D3BJ/DZVP. Different colors/styles compare different OpenFF versions beginning with version 1.0, and for reference, GAFF 2.11 with AM1BCC charges is shown for comparison. The inset zooms in on the boxed portion of the CDF. Adapted from ref (112). Available under a CC-BY 4.0 license. Copyright Mobley, Wagner, Wang and the Open Force Field Initiative, 2023.

In the Sage series, our OpenFF 2.1 release made a number of further improvements. Previously, the OpenFF objective function had optimized torsional parameters primarily based on torsion drive data, ignoring dihedral deviations used in optimized geometries. We updated our fitting process to include dihedral deviations observed in optimized geometries, resulting in improved performance on validation data sets (Figure 4). We also incorporated new initial guesses of parameters derived from the modified Seminario method (discussed further below) which improved accuracy and gives more physical values for valence geometries and force constants. We also began to fit improper torsion parameters, observing significant benefits for molecular geometries, as observed by RMSD and torsion fingerprint deviation while maintaining generally good energetics.^112^ Overall, these improvements improve accuracy relative to our QM benchmarking data substantially.

Overall progress across several OpenFF force field versions (on benchmark data, not training data sets) is shown in Figure 4 for RMSDs. Additional benchmarking data is available, including in internal industry tests on proprietary chemistries.^79^

### 2.2 Advances in Science

#### 2.2.1 Training Data Selection

As mentioned, a key distinction of OpenFF force fields is their broad coverage of chemical space with relatively few parameters. The selection of data to use in parameters fitting is crucial for the quality of the results. Important choices include the balancing between data set size and the breadth and depth of coverage of chemical space, as well as which properties to use as targets.

The Parsley 1.2.0 force field was fit to a high-level QM training data specifically curated with the goal of increasing chemical diversity and parameter coverage. Recent version of Sage line of force fields, Sage 2.1.0, used optimized conformers of less than 1600 unique small molecules and one-dimensional torsion scans of less than a 1000 unique small molecules. Even with these smaller curated sets of molecules, which represent the pharmaceutically relevant chemical space, the performance is comparable to other small molecule force fields and ML potentials.

The QM data used by OpenFF in force field fitting is generated using the B3LYP-D3(BJ)/DZVP method and basis set. This choice^113^ was initially based on benchmark studies of conformer energies of neutral peptides and macrocycles,^114,115^ as well as a comparison of relative torsion profile energies on a set of 15 one-dimensional torsion scans. We recently carried out a more comprehensive benchmark of 20 combinations of functionals and basis sets across Jacob’s ladder of chemical accuracy, comparing relative energies across torsion profiles and dipole moments.^86^ The data set considered 59 molecules selected to represent chemical diversity, including variations in central bonds, formal charges, elements, and intramolecular interactions. As such, this data set included molecules with nonzero formal charges, strong internal interactions, conjugated central rotatable bonds, and halogens. Gratifyingly, we found that the B3LYP-D3(BJ)/DZVP level of theory yielded the best balance between computational expense and the accuracy necessary for force field fitting.

Likewise, we carefully considered which properties to use in retraining Lennard-Jones (LJ) parameters. Historically, LJ parameters are often trained to experimental physical properties, commonly a combination of density and heat of vaporization (*ΔH*_*vap*_) measurements.^116−118^ However, issues such as low availability of *ΔH*_*vap*_ data and the necessity of simulating two different polarization states (i.e., the liquid and gas phases) with the same fixed charge force field complicated training, to *ΔH*_*vap*_ in particular.

OpenFF has so far focused on training to mixture data such as densities of mixtures and enthalpies of mixing. Liquid mixture data do not require simulating in multiple phases, or including/correcting for effects of changes of polarization upon transfer between phases. More specifically, the change in polarization on phase change (gas-to-solution, such as gas-to-water) is much larger than the change in polarization on transfer between solvents (e.g., water to a nonpolar solvent), so transfer within the same phase requires fewer corrections than transitions across phases. Moreover, mixture properties can allow the addition of a range of complexity into the training data; mixture properties can capture interactions between the two components that pure properties cannot, across a range of different compositions, which is much more like the environment encountered in simulations of biomolecules at realistic conditions. Finally, many more data points are readily available for mixture properties than for pure ones. We compared training LJ parameters to four combinations of pure and mixture physical property data in order to systematically compare the performance of training a force field to those data sets.^87^ We found that training to mixture property data resulted in statistically significant improvements on benchmarks such as solvation free energies over training to pure liquid (or phase change) data only. The LJ parameters in Sage 2.0.0 were therefore trained to a mixture data set of densities and enthalpies of mixing, resulting in improved performance on both aqueous and nonaqueous solvation free energies relative to the previous Parsley 1.3.0 force field.

#### 2.2.2 BespokeFit

As discussed, one of the benefits of the OpenFF direct chemical perception approach is the compactness of the resulting force fields. For example, the Sage force field contains 167 torsion parameters, in comparison to around 150 K such parameters in the OPLS3e library,^119^ with small or negligible differences in accuracy over large scale protein–ligand binding tests.^120^ Torsion parameters, however, might be expected to be less transferable than other valence parameters, since they must account for a range of stereoelectronic and steric effects, and there are numerous examples in the literature of discrepancies between the potential energy surfaces of classical force fields and quantum mechanics.^121,122^ To complement the transferable force field libraries, we have therefore developed OpenFF BespokeFit as a tool to automate the optimization of custom torsion parameters against reference quantum chemistry data for SMIRNOFF-style force fields in collaboration with the Cole group at Newcastle University.^52^

Custom parameter assignment can historically be a time-consuming and error-prone task, but OpenFF BespokeFit automates all stages from molecular fragmentation to reference data generation and parameter optimization. Fragmentation of larger molecules can reduce the cost of quantum chemistry reference calculations, and reduce the risk of hysteresis in the energy profiles. It is important not to oversimplify the chemical environment, however, and we have found that the Wiberg bond order is an effective surrogate measure of the disruption of the potential energy surface caused by a proposed fragmentation.^123^ Once the rotatable bonds for reparametrization are identified, BespokeFit makes use of the TorsionDrive package with wavefront propagation and geomeTRIC for performing one-dimensional torsion scans.^124,125^ Importantly, an interface with the QCEngine package^126^ offers access to a suite of quantum chemistry, semiempirical and machine learning potential reference data methods through a single interface. In keeping with the OpenFF open science philosophy, we have additionally developed QCSubmit as a tool for scheduling quantum chemistry data sets at-scale, and aggregating the results for storage on public (such as QCArchive^96^) or private repositories.

Using QCSubmit to curate the calculations, and the ForceBalance software^56^ to optimize the torsion parameters, we have demonstrated the utility of BespokeFit in deriving custom parameter sets for a large data set of 490 molecular fragments.^52^ Across this set, the root-mean-square error in the potential energy surface, relative to the quantum chemistry reference, was reduced from 1.1 kcal/mol using the baseline, transferable force field, to 0.4 kcal/mol with BespokeFit. Importantly, we have also shown that the resulting force fields can yield benefits in alchemical free energy calculations. Correlation between theory and experiment for a set of 16 congeneric inhibitors of the TYK2 protein was increased from 0.72, using the baseline force field, to 0.93 using the bespoke version.^52^ We also saw encouraging accuracy, intermediate between the baseline force field and full QM, using the computationally less expensive GFN2-xTB semiempirical method^127^ to generate the reference potential energy surfaces. Whether these observations hold across a wider range of protein targets and ligands will require further benchmarking. A subset of this BespokeFit workflow has been implemented within the Cresset Flare software,^128^ and since publication OpenFF-BespokeFit has been used to parametrize a custom force field to study possible degradation mechanisms in modified nucleic acids.^129^

#### 2.2.3 Using the Modified Seminario Method for Initial Values

Force field fitting must begin from some initial guess of parameters, especially as the force field must cover diverse chemistries and be fit to a variety of different physical and computed properties. Equilibrium bond lengths and angles can be estimated to be close to the mean reference values of the training set of molecules, but bond and angle force constants are difficult to obtain from molecular geometries alone. The optimizer may get stuck in local minima if the starting point is far away from the global minimum resulting in physically nonsensical force constants such as double bonds having a lower force constant than single bonds. The modified Seminario method^130^ utilizes the QM Hessian data of the training set of molecules. By projecting the eigenvectors of partial Hessian matrices onto bond and angle vectors for the atoms involved, the method enables the selection of initial bond and angle force constants. These parameters have been shown to closely reproduce quantum mechanical normal-mode frequencies,^130^ and provided us with physically relevant bond and angle parameters that enabled us to significantly improve the force field. Table 1 shows the stark difference in bond parameter force constants with using a modified Seminario starting point. This can also help in choosing appropriate priors that may keep parameter values closer to the initial values during optimization. This automated choice helps remove the human element in picking the right initial force constant for bonds and angles.

**Table 1:** Bond Force Constants Are Physically More Intuitive with Starting the Force Field Fitting from the Modified Seminario Method Estimated Values for Bond and Angle Parameters^a^

	Sage 2.0.0	Sage 2.1.0
Bond SMARTS	*k* (kcal/mol/ang^2^)	*k* (kcal/mol/ang^2^)
[#7X2:1] – [#7X2:2]	675	473
[#7:1] – [#7:2]	845	578
[#7X3:1] – [#7X2:2]	830	620
[#7:1]:[#7:2]	732	662
[#7:1] = [#7:2]	698	1089
[#7 + 1:1] = [#7 – 1:2]	766	2440
[#7:1]#[#7:2]	760	3237

a The single bonded, aromatic, double bonded and triple bonded nitrogen bond parameters are listed in ascending order of force constants as expected from QM in Sage 2.1.0, when compared to Sage 2.0.0 values. While the Sage 2.0.0 values are the legitimate result of a fit, they appear unintuitive, especially for triple bonds, likely because the optimizer pushed them to those values since in multi-objective optimization there can be a number of solutions reaching the same minima.

The choice of what type of properties to use in training is also important. Removing vibrational frequencies as a training target in Sage 2.0.0 improved accuracy over previous generations of Parsley since the difficulty in correctly matching the vibrational modes between QM and MM was avoided. In the Sage 2.0.0 and 2.1.0 releases, valence parameters were trained only to a combination of optimized geometries and torsion energy profiles and a careful application of priors eliminated previously observed pathologies such as errors in sulfonamide angles.

#### 2.2.4 Probing Alternative van der Waals Functional Forms

Historically, choices in force field design strategy are made early on and become “baked in”, typically for many decades. One such example is the choice of the Lennard-Jones 12–6 potential that describes nonbonded repulsion at short-range and attractive dispersive interactions at longer range. The functional form of this potential is not entirely physically consistent;^131,132^ while the *r*^–6^ term was chosen to model the physical shape of the long-range attractive tail, the repulsive *r*^–12^ potential was chosen in part due to computational motivations, as it is simply calculated by doubling the *r*^–6^ term.^133^ However, it has persisted because rewriting molecular modeling software and refitting force fields to test other functional forms would previously have been impractical. To enable easy optimization of parameters using novel van der Waals functional forms, we wrote Smirnoff-plugins as an interface to extend the OpenFF software stack to support custom nonbonded functional forms.^36^

As a proof-of-concept, we experimented with replacing the Lennard-Jones potential with a double exponential (DE) functional form,^134^ which has a physically motivated exponential decay at short-range and an additional parameter to control the decay of the attractive potential at long-range. Using the Smirnoff-plugins interface to the OpenFF stack, we cotrained a DE-based small molecule and water transferable force field (DE-FF) on over 1000 physical properties.^36^ As well as improvements over the LJ-based Sage force field on the training set, we also saw improved metrics when DE-FF is benchmarked on transfer free energies (RMS errors of 0.85 kcal/mol, *r*^2^ = 0.93). In this way, new force field hypotheses can be made and tested in a matter of weeks, rather than the years of human time that would have been required before OpenFF.

This project represents an early demonstration of our community building efforts. Our infrastructure drives community innovation by enabling the rapid prototyping, implementation, and derisking of ideas before they are brought into full production. For example, if the proof-of-concept work on DE-FF force fields continues to show value, this may drive mainline force fields to move in this direction, as their natural soft core and promising accuracy has the potential to benefit free energy calculations.

#### 2.2.5 Automated Chemical Perception Using Binary Encoded SMARTS

Since the original description of the SMIRNOFF format,^135^ a key motivation has been to automate direct chemical perception using SMARTS patterns^78^ to extend and build general small-molecule force fields. The difficulties associated with extending small-molecule atom types has been previously discussed.^75^ The use of SMARTS patterns as the perception model in the SMIRNOFF format simplifies the process of adding new parameters. However, experience has shown that determining general SMARTS patterns by hand can still be very difficult. We have seen some success with designing SMARTS patterns that are specific to relatively narrow chemistries to help avoid specific pathologies; for example, we introduced new SMARTS for amides in 1.3.0 and sulfonamides in 2.1.0. In the 1.3.0 release (see the release notes at https://github.com/openforcefield/openforcefield-forcebalance/releases/tag/v1.3.0) we investigated an energy cusp in the torsion profile of N-methyacetamide, resulting in MM disfavoring the flat conformation, whereas the QM reference torsion profile indicated that the flat conformation was stable. The problem was fixed by splitting the SMARTS patterns t69 and t70 in 1.2.0 to t69 and t69a, and t70 to t70b-d. Examining these splits show that 69a splits off torsions for diakyl amides, R—C(=O)N(R′)(R′′), from 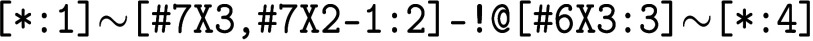 (a torsion linking (trivalent nitrogen or negatively charged divalent nitrogen) by a nonring bond to a trivalent carbon) to 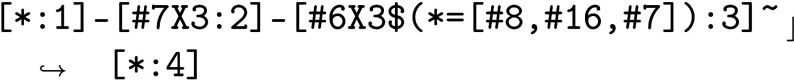 which is a torsion linking a trivalent nitrogen to a trivalent carbon that is connected to an oxygen, nitrogen or sulfur.

Following this, the parameter t70 was split to differentiate the pattern 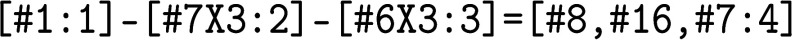 (a torsion linking central atoms consisting of a trivalent nitrogen, single bonded to a trivalent carbon, where the carbon is double-bonded to a nitrogen, oxygen or sulfur) to the three more specific patterns 
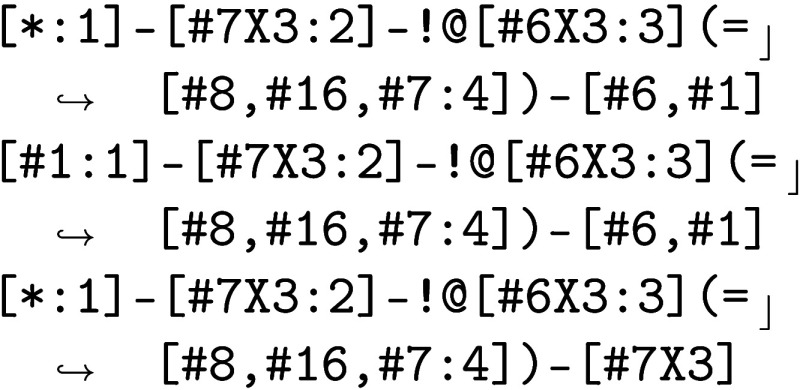
 which specify that the central bond is a nonring bond and specify the identity of other atoms involved in the torsion or adjacent to the trivalent carbon. The first and second patterns specify that the other atom connected to the trivalent carbon is a carbon; in the third pattern, it is a nitrogen. In the second pattern, the first of the four atoms involved in the torsion is a hydrogen.

A subsequent parameter fit with these new parameters successfully corrected the cusp. In the newly split SMARTS patterns for t70, for the third atom, the nonring carbon, the neighbor atoms were made more specific covering the cases of R in R—C(=O)N(R′)(R′′) being a carbon, hydrogen, or a nitrogen. And, the difference between the first and second splits was in the first atom being a wild card and a hydrogen, respectively. It is to be noted that the parameter t69 is still the most general with wild cards for atoms 1 and 4, and subsequent specific parameters were introduced below it according to SMIRNOFF hierarchy, where the most specific parameter gets assigned over a general parameter. In the 2.1.0 release, we modified an angle SMARTS pattern to fix distorted sulfonamide geometries (see the release notes at https://github.com/openforcefield/sage-2.1.0). To fix this, we modified angle parameter a32, represented by the SMARTS pattern, 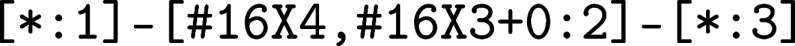 which specifies a generic angle (involving single bonds only) around a tetravalent sulfur or trivalent neutral sulfur, to 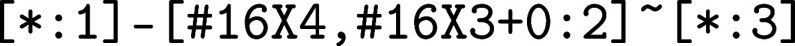 to separate [*]-[S]=[*] from [*]=[S]=[*]. Notice the bond change between the second and third atoms from a single bond to any bond represented by tilde. Prior to this fix, O=S=O and N–S=O used to get the same parameter and N–S–N has a separate parameter. In this case, the O=S=O equilibrium angle was generally 120 degrees while the others were near 100 degrees. The subsequent fit with this modified SMARTS pattern was able to fix the distorted geometry. It is important to note that these SMARTS patterns were developed by a human expert and the final improvements came through after a number of iterations of human-in-the-loop development.

Because of the complexity of deriving patterns by hand, we are extremely interested in ways to automatically sample SMARTS patterns, but finding a solution has thus far proven difficult. ChemPer was developed to sample SMARTS using a Monte Carlo algorithm and works by partitioning a group of molecular fragments into two or more groups/parameters.^81^ However, this random sampling approach proved to be too computationally expensive for general force field development, as the sampling is done on an exponentially growing chemical space. Additionally, there are many ways to write a SMARTS pattern to match the same substructures, meaning that the search space is highly redundant. Regardless, the ChemPer approach has been a valuable tool and is heavily used in BespokeFit for determining SMARTS patterns that isolate individual torsions for custom parameter fitting.

We have laid out a theoretical framework for sampling SMARTS patterns in a direct, iterative manner using binary-encoded SMARTS (BESMARTS).^136^ ChemPer seeks to find SMARTS patterns that discriminate between specific groups of molecules or chemistries, whereas BESMARTS inverts the problem and instead provides a list of rapidly computable SMARTS that each induce a partitioning of the input data, then determines which of these partitionings are useful. These SMARTS patterns can be individually evaluated as candidate parameters in a force field fit, and highly performing candidates (evaluated based on which proposed new parameters results in the largest improvement in the objective function) can be included in the chemical perception model.

**Figure 5:**
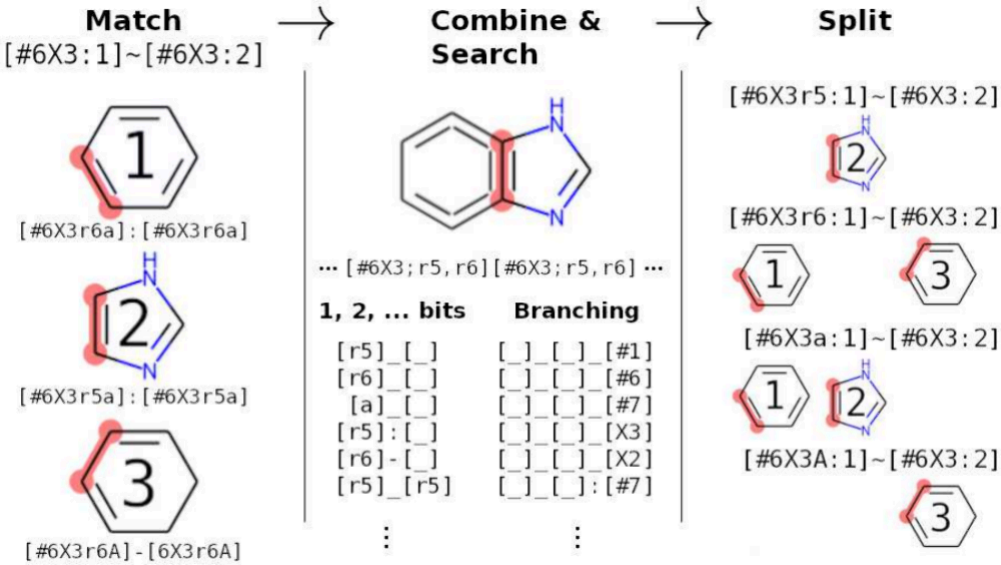
General approach of BESMARTS parameter search. For each parameter, the chemical environments that matched are combined into a single pattern. The combined pattern identifies SMARTS primitives that have multiple values that are then used to derive new patterns. Each new pattern is based on the original parameter ([#6X3:1] [#6X3:2]) with one or more primitives (represented as bits) added. In this example, the bonds that matched, when combined, show that bonds in 5-membered rings and 6-membered rings matched the original parameter. This offers the r5 and r6 primitives as a means to split, and the new candidate parameters [#6X3r5:1] [#6X3:2] and [#6X3r6:1] [#6X3:2] are generated and subsequently evaluated for performance. The splits can take multiple bits simultaneously, can additionally search the local environment for additional primitives to find more specialized splits. Image adapted from Gokey and Mobley.^136^ Available under a CC-BY 4.0 license. Copyright 2023, Gokey and Mobley.

Because the BESMARTS approach searches SMARTS patterns in a breadth-first-like search, candidate SMARTS are as general as possible while still pinpointing salient molecular features that are important for improving force field performance. For example, a list of potential candidate splits from a generic sp^3^ carbon bond ([#6X4]-[#6X4]) could be [#6X4H3]-[#6X4] (a tetravalent carbon with three protons connected to another tetravalent carbon), [#6X4r]-[#6X4] (a tetravalent carbon in a ring connected to another tetravalent carbon), and [#6X4H0]-[#6X4] (a tetravalent carbon with no protons connected to a tetravalent carbon). While these are equally general in terms of SMARTS, [#6X4r]-[#6X4] (which specifies the first carbon is in a ring) would split out cyclic molecules and possibly lead to a better parameter over the other two candidates, especially if this process is applied to torsions (Figure 5). Automating this type of search should facilitate the further development of general, small-molecule force fields.

We have used these concepts and an in-house, prealpha BESMARTS implementation as a copilot to guide the development of the sulfonamide patterns found in OpenFF 2.1.0. Additionally, early applications of the automated parameter search also uncovered areas in our small-molecule force field that could be extended due to multimodal distributions in the underlying QM geometries; in particular how we treat bond and angle parameters for 3-, 4-, and 5-membered rings. This was accomplished by generating various SMARTS patterns and looking at the mean equilibrium bond/angle values for each split SMARTS, where ring systems were found to have substantially different mean values. Importantly, these differences can also be found in examining the bond and angle force constants for such rings versus their linear counterparts. Such mixing of different chemical moieties in a force field fit can be problematic if there is an uneven distribution of data where the parameters drift toward the dominating case, or worse if the distribution is multimodal and the parameters optimize to some unreasonable mean value as a best-effort compromise. In an automated parameter search, multimodal cases can be discovered and fixed as each split will subsequently fit to each mode in the data distribution individually, leading to an accurate set of parameters.

Due to the complexity and variety of edge cases involved when manipulating SMARTS patterns at the detailed level needed for force field parameter search, our BESMARTS implementation is still under heavy development at https://github.com/trevorgokey/besmarts. The design of BESMARTS is targeted to the general case of clustering molecules by SMARTS patterns, and as such should be useful not only to force field chemical perception modeling, but to any application requiring a clustering method that labels a group of molecular fragments with an aggregate SMARTS pattern. For example, it is possible to generate a list of SMARTS that mimic the chemical perception model of non-SMARTS based methods. Two interesting examples where this could be applied are atom-typed models, such as GAFF, where a mapping of SMARTS to types is sought, or even ML potentials, where SMARTS patterns are sought which cluster parameters such as bond force constants that are similar in value. Creating SMARTS models for such examples can be helpful to describe how their underlying chemical perception models work and provide a method to compare force fields with otherwise disparate chemical perception models using the common language of SMARTS.

### 2.3 Infrastructure Advances and Interoperability

#### 2.3.1 OpenFF Toolkit

The OpenFF Toolkit is the central library in OpenFF’s software infrastructure. It provides a reference implementation of the SMIRNOFF specification. It is written in Python and is primarily distributed as a conda package. Documentation, including installation instructions, is available online at docs.openforcefield.org. This page also provides several Jupyter notebooks showcasing core functionality that can be run in-browser on Google Colab.

Broadly, the OpenFF Toolkit focuses mainly on parsing SMIRNOFF force fields and applying SMIRNOFF force fields to chemical topologies. However, it also provides utilities to manipulate force fields, as well as to bring molecules in from standard cheminformatics toolkits and export parameters suitable for simulation in several standard modeling tools.

For most users, the primary use of the OpenFF Toolkit is to apply an existing force field to their molecules of interest and then to run a simulation. The OpenFF Toolkit aims to make this use case as streamlined as possible, and automatically handles complex tasks like partial charge assignment, SMIRKS based matching, and simple molecule sanitization. The output of a standard user workflow using the OpenFF Toolkit is an OpenMM System object, which can easily be used to begin a simulation. More complex workflows, including conversions to and from other ecosystem formats, can be made using the OpenFF Interchange object.

The toolkit is based around a Molecule class with a rich internal representation of a molecule. Molecules can be loaded from a variety of sources into OpenFF Molecule and Topology objects. Currently, these sources include file formats like MOL/SDF, SMILES, and some PDB files. Additionally, the OpenFF Toolkit interfaces with the RDKit and OpenEye Python APIs and can interconvert with molecule representations in those packages.

An OpenFF Molecule is a graph representation of a molecule that consists of, at a minimum, atoms defined by element, formal charge, and stereochemistry, and bonds defined by integer bond order and stereochemistry. These are the minimum pieces of information needed to perform SMIRNOFF parameter assignment. Optionally, there may be additional information stored on Molecules, such as conformers, partial charges, and name, and on Atoms, such as name, residue, and chain.

The OpenFF Molecule class includes methods for input from and export to common file formats and data models such as SDF files, SMILES patterns, or QCSchema models used by QCArchive. There is a high-level API for common chemical operations such as partial charge assignment and conformer generation. These operations are handled by wrappers around existing toolkits (currently OpenEye Toolkits, the RDKit, and AmberTools).

The OpenFF Toolkit now includes first-class support for loading biopolymers, including loading proteins from PDB files. Previous versions lacked this functionality because PDB files lack chemical information required for OpenFF’s representation of molecules, which include bond order and stereochemistry. To bridge this gap, a library of known chemical substructures is matched against residue metadata in PDB files and added to the internal representation of the PDB file after loading. The first release (version 0.11.0) only supported loading single peptides in vacuum, but the current release 0.13.0 introduced Topology.from_pdb for loading multicomponent PDB files. This enables interoperability with more complex PDB files containing multiple proteins, multichain proteins, solvent and crystal water, common ions, and ligands, if also provided extra information such as an SDF file or SMILES pattern.

The OpenFF Toolkit has three primary areas intended for easy extension by outside developers:

- The ToolkitWrapper plugin interface registers wrappers around external cheminformatics toolkits. For example, calls like Molecule.generate_conformers() perform a complex task on native OpenFF objects by delegating to external toolkits such as RDKit or OpenEye, while keeping the ecosystem-specific details of the object conversions away from the user. Currently, OpenFF provides wrappers around important functionality from AmberTools, RDKit, and OpenEye. External developers can create custom ToolkitWrapper classes and add them to the global registry at runtime, where they will automatically be used for background cheminformatics operations by various parts of the OpenFF Toolkit.
- The ParameterHandler plugin interface allows for parsing of nonstandard fields from a SMIRNOFF-format force field, enabling experimentation with different functional forms such as nonharmonic bonds or non-Lennard-Jones nonbonded interactions. Plugins are also free to change how parameter assignment is performed, allowing experimentation with alternatives like machine-learning based parameter assignment or the creation of virtual sites.
- The smirnoff_force field_directory Python entry point can be extended by any Python package and enables discoverability of additional SMIRNOFF force fields. This entry point is intended to enable anyone to distribute SMIRNOFF force fields as Python packages such that they are immediately loadable by the OpenFF Toolkit, without requiring users to run separate downloads or manage relative paths.

#### 2.3.2 OpenFF Interchange

The toolkit provides an API for interacting with SMIRNOFF force fields, molecules, and topologies composed of multiple molecules. OpenFF Interchange is a data model and Python package that captures the state resulting from applying a force field to a topology. From here, one can export to common simulation engines like OpenMM, GROMACS, Amber, and LAMMPS. Interchange is not limited to only SMIRNOFF force fields; it currently provides an interface from Foyer force fields^138^ and can also import existing systems prepared with other force fields and stored in OpenMM or GROMACS files. It fully supports the features of the SMIRNOFF specification, including features not yet present in mainline OpenFF force fields, such as virtual sites, biopolymer parameters, implicit solvent interactions via GBSA, and WBO-interpolated valence parameters. Its API provides robust access to all interaction parameters contained in a system, which enables more advanced features such as exports to vectorized representations as are useful in machine learning optimization approaches. With the 0.11.0 release of the OpenFF Toolkit, OpenFF Interchange is now used as the backend for creating OpenMM systems.

#### 2.3.3 OpenFF Evaluator

In addition to fitting against large quantities of high-quality quantum chemistry data, OpenFF’s force fields are also fit and benchmarked against condensed-phase physical property data. These calculations are orders of magnitude slower than single-molecule geometry optimizations and necessitated the development of an automated approach. OpenFF Evaluator^87^ is a fully automated, highly scalable framework for evaluating physical properties and their gradients. It is released as a Python package which handles parsing data from experimental databases, running molecular simulations, caching simulation data, estimating physical properties via a multiscale approach, and computing gradients of these properties with respect to force field parameters. It was used for fitting vdW parameters in both Parsley^85^ and Sage^39^ and has been used to benchmark these force fields against each other and some versions of GAFF. It supports estimating liquid density, enthalpies of vaporization and mixing, dielectric constants, excess molar volumes, solvation free energies, and host–guest binding free energies. The Python API supports plugins which can enable the estimation of other physical properties as well.

### 2.4 Science Advances Enabled by Open Force Field Infrastructure

#### 2.4.1 Tuning Potential Functions to Host–Guest Binding Data

Although a prime application of simulation force fields is the prediction of small molecule-protein binding affinities (i.e., binding free energies) for drug discovery, the experimental data sets typically used to adjust force field parameters do not include binding free energies, and protein–ligand binding free energies are generally still too computationally costly to include in a parameter optimization cycle. However, in recent years, calculations of the standard (or “absolute”) binding free energies (ABFE) of host–guest systems have grown more efficient and automated, and these systems are compact enough that one can integrate them into the force field parameter optimization loop.

We demonstrated the feasibility of this tactic by using OpenFF infrastructure and a data set of 126 aqueous host–guest systems, spanning cyclodextrins, cucurbiturils, and deep cavity cavitands, to retrain and test a generalized Born implicit solvent model.^139^ To accomplish this, we implemented a feature in OpenFF Evaluator^87^ to estimate host–guest ABFEs. Initial benchmarks against experiment, using the Sage force field and a generalized Born implicit solvent model, showed that the binding free energies were grossly overestimated, particularly for cucurbituril complexes, with RMS errors on the order of 20 kcal/mol. We then used OpenFF Evaluator and ForceBalance to optimize five generalized Born cavity radii against a host–guest training set, and found that the optimized radii performed extremely well on the test set, with RMSE falling to about 2 kcal/mol. The trained GB parameters also markedly reduced the tendency of the model to overestimate protein–ligand binding free energies in a separate test set.

However, this study surfaced a hitherto unknown trade-off in generalized Born parametrization between getting binding free energies right and getting hydration free energies right, because the cavity radii that give accurate binding free energies lead to overestimation of hydration free energies i.e. they were too negative. It is thus of high interest to develop implicit solvent models that are more globally applicable and therefore more transferable and accurate. This work also sets the stage for potential future use of host–guest binding data to adjust nonbonded parameters such as Lennard-Jones σ and ϵ in the context of the explicit solvent models typically used in protein–ligand binding free energy calculations.

#### 2.4.2 A Fast, Convenient, Polarizable Electrostatic Model for Molecular Dynamics

The force fields most widely used for biomolecular simulations—including current OpenFF versions—do not include an explicit treatment of electronic polarizability, but instead handle it implicitly, through empirical adjustment of other parameters. This approach has worked quite well for many years, but is still expected to limit accuracy, especially in settings where the electrostatic fields felt by molecules change markedly in the course of a simulation and thus polarize them to a greater or lesser degree. Important progress has been made in integrating explicit representations of polarizability into force fields,^35,55,140−143^ but nonpolarizable force fields are still used much more widely, presumably because the improvements in accuracy polarizable force fields provide have not seemed consistent or large enough to merit the associated increase in computational cost. In addition, there are few tools to assign polarizable force field parameters to new molecules.

Wang and co-workers have now used OpenFF data sets and capabilities to prove the principle of a facile approach to including electronic polarizability in simulations, with the goal of gaining much of the potential increase in accuracy at a modest computational cost.^144^ The method includes a set of typed polarizability parameters; i.e., atom-centered point polarizabilities that are assigned to a new molecule based on its 2D structure, rather than by using bespoke QM calculations. These polarizabilities were fitted to changes in the QM electrostatic potentials (ESPs) of a training set of molecules, and have been typed both by element and by LJ type. It also includes a new set of bond-charge corrections (BCCs) that, when combined with a traditional population-based AM1 partial charge assignment, yields a final set of charges that integrate properly with the polarizabilities in the sense of generating accurate QM ESPs around training-set molecules. This framework thus allows facile assignment of a polarizable electrostatics model to a new molecule of interest. The model comprises atom-centered partial charges (generated by AM1 and the tuned BCCs) and typed atom-centered point-polarizabilities. In addition, for the sake of computational speed, the model was parametrized using the direct approximation,^145^ in which the point-polarizabilities feel only the partial charges, not the induced dipoles on other atoms. This work made use of optimized molecular geometries from the OpenFF BCC Refit Study COH v2.0 data set to compute QM ESPs at the MP2 level on suitable grid points using the OpenFF Recharge library. The BCC types used the same SMARTS patterns as used by the original AM1-BCC method, and the BCCs were trained with a version of the OpenFF Recharge package that was modified to handle polarizability.

The polarizabilities and BCCs were obtained by training against a subset of the OpenFF ESP Fragment Conformers v1.0 data set. The resulting electrostatics model, termed AM1-BCC-dPol, used new BCCs trained with OpenFF Recharge. The multipole and induced dipole (MPID) SMIRNOFF plugin supports simulations with AM1-BCC-dPol in OpenMM, and thus allows parametrization of polarizable OpenFF force fields via the OpenFF Toolkit or OpenFF Interchange and comparisons with experiment via OpenFF Evaluator. AM1-BCC-dPol has given encouraging results in initial benchmark studies, even without further adjustment of e.g. LJ parameters to match it. In particular, AM1-BCC-dPol maintained the accuracy of densities of organic liquids and provided a marked improvement in the accuracy of their dielectric constants.^144^ Planned further studies of this approach include development of a water model and retraining of Lennard-Jones parameters within this paradigm, making it possible to provide quantitative evidence for what regimes might require treatment of polarizability for accuracy.

#### 2.4.3 Parameterization of General Organic Polymers within the Open Force Field Framework

One significant issue with parametrization schemes for small molecules is extending them to larger polymers. Two main issues with loading and parametrizing polymers with force fields is that the output of many polymer building tools do not contain as much chemical information as is generally contained in small molecule workflows. We have recently developed and published a schema^146^ for reading in PDB (or other coordinate file formats) for long polymers, and inserting in the necessary information such as bond order and formal charges using customizable monomer templates based on SMARTS strings. With this information injected into the molecular topology, the entire polymer system can then be easily parametrized using the existing OpenFF toolkit. This process is essentially a generalization of approaches typically used for loading proteins from PDBs and has been tested and validated over dozens of polymer systems with a wide range of chemical functionality and sizes.

Another key problem with parametrization of polymers is that the methods for determining partial charges, such as AM1-BCC, scale extremely poorly for larger molecules, potentially taking hours for molecules of more than 100 heavy atoms, and thus becoming essentially unusable. The standard approach is to develop template charges which is a relatively intensive manual process. We have developed a workflow along a two-pronged approach.^146^ First, we have developed a workflow for producing such template charges for monomers within polymers. Although producing charges very close to full AM1-BCC for long oligomers, such a process is still relatively slow and occasionally prone to errors. As a longer term solution, we have developed tools to incorporate graph neural network charges, initially with espaloma-charge(147) and the in-house OpenFF NAGL package (https://github.com/openforcefield/openff-nagl), described below, which reduces the time to determine partial charges of a large polymer system to seconds. Although our initial testing indicates a small amount of additional tuning may be necessary to make such partial charging schemes as accurate as AM1-BCC, the rapid advance in such approaches suggests that they will be a useful and perhaps the best option in the very near future.

Overcoming the obstacles to parametrizing polymers within the OpenFF framework opens the door to use OpenFF to investigate a range of biotechnological applications such as small molecule interactions in polymer formulations, proteins with noncanonical amino acids, proteins with post-translational modifications, nucleic acids with chemical modifications, and polymer–protein conjugation, as well as a wide range of soft material science applications. Additional data on validation and examples for polymer templates can be found at https://github.com/openforcefield/polymer_examples), with further OpenFF-based polymer setup tools being developed at https://github.com/shirtsgroup/polymerist.

#### 2.4.4 Graph Neural Network-Based Force Fields

One of OpenFF’s key developments has been the reenvisioning of the perception of local chemical environments in order to better assign atomistic parameters via direct chemical perception. But there may be limits to how well discrete atom types can match the accurate molecular diversity, and optimization of a discrete space in parameter types is significantly more complicated than optimization of parameters themselves. OpenFF has been investigating ways to further expand the perception of molecular environments in assigning parameters.

Graph neural networks—neural models aggregating and updating node (atom) and edge (chemical bond) representations in a permutation-invariant manner^148,149^—can play a similar role to molecular mechanics atom typing schemes.^61^ As such, they can replace discrete, human-derived atom embeddings with continuous representations, avoiding the need for exponentially more discrete atom or parameter types, as increasingly precise interaction parameters are required. Espaloma,^60,61^ for example, designed using the infrastructure and data pipeline of Open Force Field, demonstrates that such a force field can be trained in an end-to-end differentiable manner to reproduce the quantum chemical energy landscape. Furthermore, although not directly trained on these targets, they also accurately reproduce quantum mechanics (QM) minima locations, NMR coupling constants, and experimental protein–ligand binding free energies.

This effort demonstrates a promising path forward for the flexible and efficient curation of an MM force field—an Espaloma-type force field takes around one GPU day to optimize from properly curated QM data, compared to tens of engineer years for the legacy, atom-typing-based counterparts. Furthermore, the gradient can flow freely to the chemical perception stage of the MM curation, enabling us, theoretically, to optimize force fields based upon ensemble observables such as physical properties.

OpenFF plans to explore the area of differentiable FFs further, as we anticipate that a wholly differentiable fitting framework will greatly improve efficiency and scalability. We are investigating how necessary certain targets are to our force field accuracy, such as fitting to optimized geometries, which remain slow even in differentiable fitting frameworks. Packages such as the recently published DMFF^64^ may further streamline fitting to challenging targets, such as physical properties. This remains an active area of research. Frameworks like Espaloma or DMFF can likely be employed to build the foundation models for MM force fields, upon which more fine-tuned versions can be tailored toward an individual user’s needs.

### 2.5 Validation against Protein–Ligand Binding Free Energies

Many industry users of force fields are motivated by the goal of accurately modeling and predicting protein–ligand interactions, so such predictions provide a key test ground for force field accuracy. At the same time, accurate calculations and predictions of binding are not solely a test of force fields, as accuracy with respect to experiment is a function of the method, system preparation, sampling, and even experimental accuracy itself.^150^ Still, benchmarking of force fields in the context of binding free energy calculations (often alchemical calculations^151^) has begun to be seen in the field as a key test and goal of force fields.

Performance on ligand-binding affinities, as with performance of any other physical observable, depends critically on the choice of benchmark set. We have worked with a diverse set of researchers to try and identify standards for choosing systems for inclusion in protein–ligand binding benchmarks,^152^ and our work in this space is open for community contributions and further refinement as the format allows for updates. This work of Hahn et al. attempts to lay out standards for inclusion, including quality of experimental data, dynamic range, availability and quality of protein–ligand bound structures, etc. However, much more work remains to be done to curate high quality binding benchmark sets, as apparent accuracy often depends as much on preparation of the system to be modeled (choice of protonation states, binding pose, etc.) as it does the force field or free energy method of choice.^151−153^ Additionally, there are still relatively few systems for which free energy calculations can be so convincingly converged that the systems serve as a true test of force fields alone.^150^ Thus, benchmarking studies, and benchmark set curation, are likely to be an ongoing process to which continual community contributions are needed.

**Figure 6:**
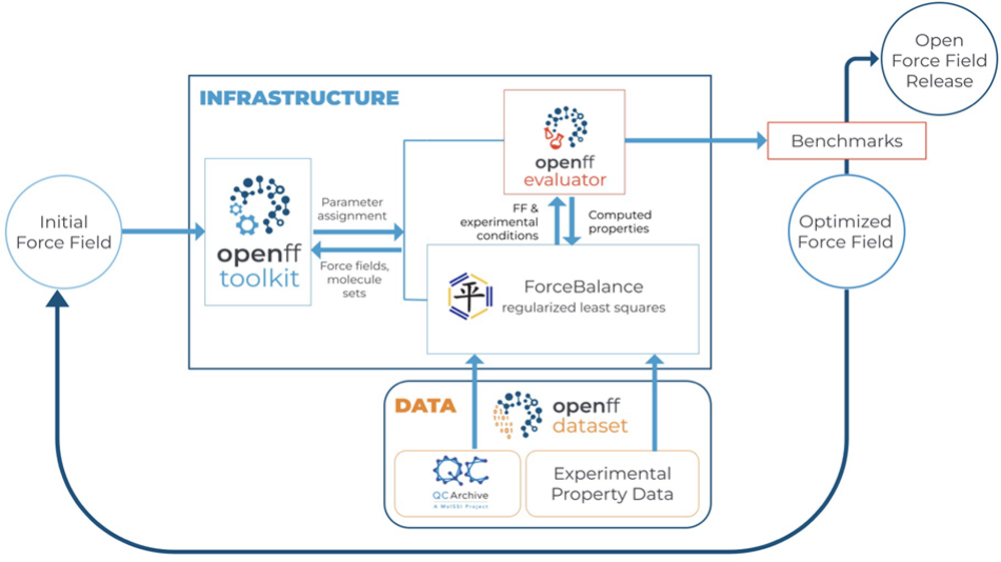
Software workflow for iterative improvement of force fields. An initial force field is implemented by the openff-toolkit, and the molecular systems needed for fitting the targeted observables are built from this force field. The force field parameters are optimized using regularized least-squares with ForceBalance, with QM data coming from stored calculations in QC Archive, and experimental condensed phase data coming from several different data sets. Condensed phase simulations are carried out using OpenFF Evaluator, and included in the optimization, though usually we optimize terms on condense phase properties after valence parameters are optimized. This produces a force field that can than then be validated. Adapted from ref (137). Available under the CC-BY 4.0 license. Copyright 2023, Boothroyd, Mobley, Wagner and the Open Force Field Initiative.

**Figure 7:**
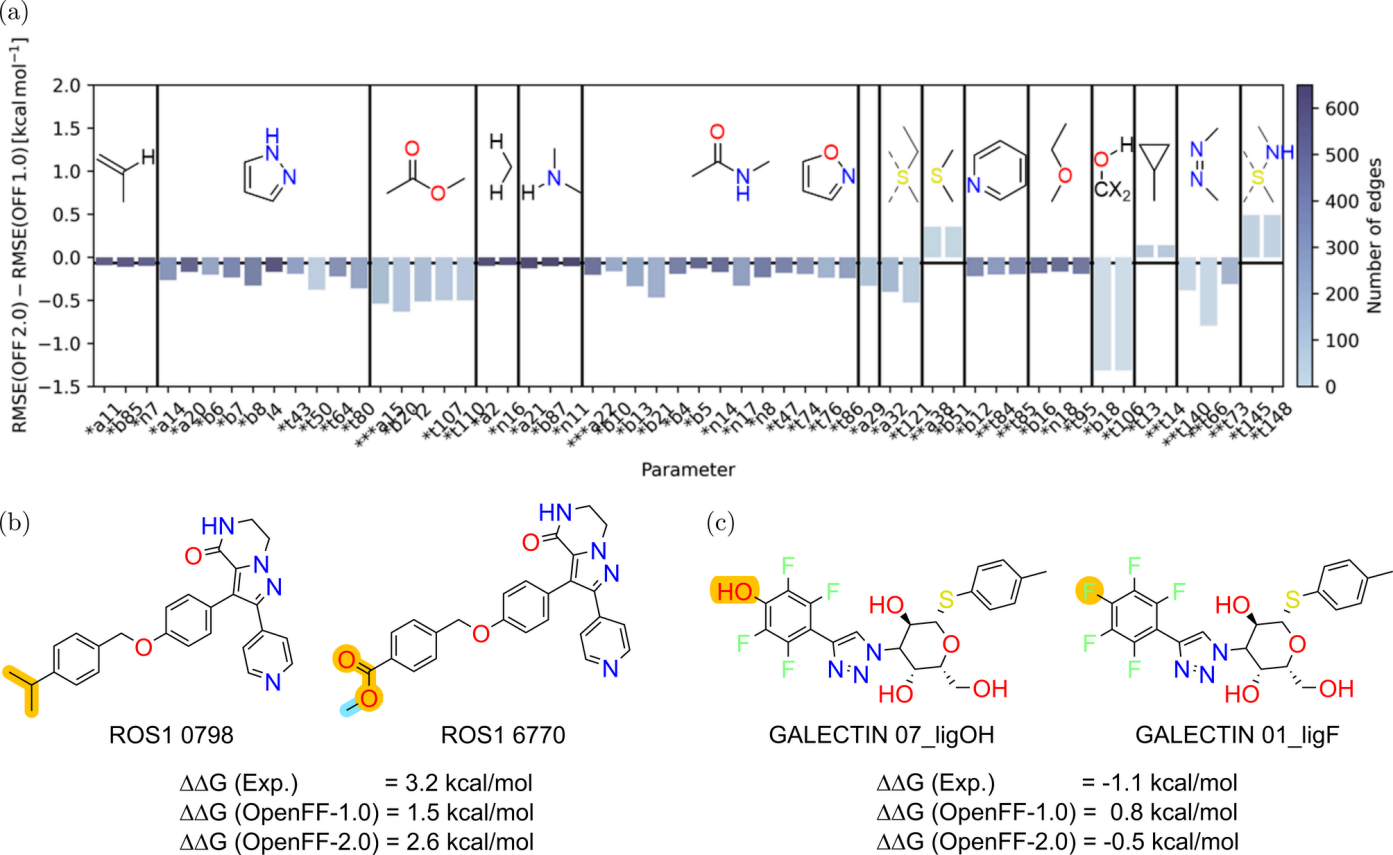
How parameter differences affect binding free energy accuracy. (a) Shown are differences in accuracy (RMS error) between OpenFF 1.0 and OpenFF 2.0, for converged relative binding free energy calculations. Only statistically significant (95% CI) changes are shown, for parameters which are used in multiple ligands across multiple targets. Stars in front of parameter identifiers indicate significant parameter changes, with more stars indicating larger changes. Upward bars indicate accuracy (relative to experiment) was decreased by the force field change, and downward bars indicate accuracy was improved. (b) and (c) show specific example relative binding free energy calculations where results changed substantially across force fields. Figure adapted from ref (153), where it is described in more detail. Available under the CC-BY 4.0 license, Copyright 2023, Hahn et al.

Given these caveats, though, several recent studies have assessed the performance of OpenFF force fields in binding free energy studies. Recent work of Hahn et al. on binding free energy calculations,^153^ and a predecessor study by Gapsys et al.^154^ assessed performance of several public force fields on a diverse set of 598 ligands spanning 22 different protein ligand targets. Overall OpenFF performed quite well among public force fields, though a consensus approach (averaging across free energy results from diverse force fields) performed slightly better. One particularly noteworthy result, however, came from cross-comparing binding free energy calculations computed across OpenFF versions, going from OpenFF 1.0 to OpenFF 2.0. In a careful test, the researchers were able to find that the change in force field version had clear effects on force field accuracy. Particularly, after significance-testing changes in binding free energies, focusing in on specific parameters which changed across versions, and narrowing the search only to parameters which were used in multiple calculations across multiple protein targets, it was possible to examine accuracy changes directly attributable to force field differences. In Figure 6 of that work, the researchers showed that most parameter changes from OpenFF 1.0 to 2.0 resulted in improvements of binding free energy accuracy, typically for specific functional groups, though roughly three functional groups actually had worse accuracy with OpenFF 2.0 (Figure 7). To our knowledge, this is the first example where significance-tested results appear to indicate clear differences across force fields, when otherwise identical system preparation and sampling is used.

Independently, a separate study focusing on a multistate method for efficient calculation of binding free energies applied OpenFF and several other force fields across four different kinases with considerable success. In a number of cases, OpenFF outperformed other public force fields, though overall they did not observe clear superiority of one force field relative to others.^155^ This seems consistent with the work of Hahn et al., who argued that in many cases, the largest errors may be due to other factors (perturbation size, inadequate input preparation (e.g., handling of missing loops or residues, selection of protonation states, selection of ligand pose, insufficient sampling) rather than force field accuracy.^153^

As the Open Force Field Initiative develops further, we will continue to work with developers of benchmarks to validate force fields for ligand binding. Although not the only measure, clear measures of performance on protein–ligand binding and other tests of real-world applications are key metrics needed to demonstrate not only the improvement of force fields, but their true utility in realistic tasks, though as noted, other factors can in some cases obscure force field accuracy.

## 3 Future Work

### 3.1 Extension of OpenFF to Protein Force Fields

The Open Force Field Initiative is actively working to develop force fields that are fitted self-consistently for simulations of *both* proteins and small molecules, including proteins with chemical modifications (such as covalently bound ligands or fluorophores) and with non-natural amino acids. Such force fields can easily—ideally with no reduction in accuracy—be applied to such “mixed” systems. Specifically, self-consistency means that the parameters assigned to a protein side chain (e.g., that of serine) will be similar to those assigned to chemically related model compounds (e.g., ethanol), and any differences will be solely due to the remaining chemical context of that group. More broadly, parameters should be assigned only based on the surrounding chemical context, not based on arbitrary human-made classifications of molecules. To sufficiently match the wide variety of protein experimental data may require additional optimization of parameters, as well as further specifications and subdivision of parameter types for the chemical environments found in proteins. However, any such changes would then be used for any small molecules with these same chemical environments as found in the biomolecules, rather than maintaining distinct parameters for protein and nonprotein environments.

Because OpenFF optimizes parameters against experimental data directly related to the noncovalent interactions of small molecules (e.g., the density of mixtures of organic liquids, and their heat of mixing with other organic liquids and water), we anticipate that such force fields will be well-suited to the key application of calculating the binding free energies of proteins with drug-like ligands. At the same time, it will be essential to check that simulations of peptides and proteins using these parameters generate conformational distributions consistent with available experimental data. As recently reviewed^156^ by a group of experts in comparison of simulated and experimental protein data, there are a number of clear opportunities for direct comparison of protein simulations to experiment. In particular, these include NMR shifts and scalar couplings of small peptides, folded proteins, and disordered proteins, which we are currently using with large scale validation tests of trial OpenFF protein force fields. In the future, we hope to also optimize or assess protein force fields via a direct comparison with crystallographic data.^157^

### 3.2 Extension of OpenFF to Other Biomolecules

In the same way that we have been working to extend Open Force Field coverage from small molecules to proteins, we have also been working to extend to other biomolecular systems and systems of biophysical significance. In the upcoming year, we hope to work on self-consistency for lipids and nucleic acids and simple ions (Group 1 and Group 2 cations, and halogen and small organic polyatomic anions), working with force field experts to develop consistent approaches, benchmark sets and parameters. Our recent developments, making it simple to parametrize arbitrary polymers,^146^ will help significantly in efforts to parametrize biopolymers such as nucleic acids and carbohydrates.

### 3.3 Neural Network Charges

Most classical fixed-charge force fields model electrostatics using static point charges centered at each atom. The electrostatic energy of the system is then typically calculated as pairwise Coulomb interactions between these partial charges. As such, generating or “assigning” well-behaved partial charges is crucial in obtaining good performance in simulation, and a number of methods have been developed to generate partial charges. One common approach is to fit partial charges to reproduce a property extracted from quantum mechanics (QM) calculations, most popularly the electrostatic potential (ESP) around the molecular surface. OpenFF force fields currently use one such method to assign charges: the AM1-BCC charge model, which produces charges by combining semiempirical AM1 population charges with bond-charge corrections that have been empirically fit to reproduce HF/6-31G*ESPs.^102^ However, AM1-BCC is victim to the flaws of many QM-based approaches: 1) poor scaling with molecular size, precluding easy application to macromolecules such as proteins, and 2) the charges generated can vary widely depending on the geometry of the conformer used in computing the ESP.

To address these issues, OpenFF plans to move toward using a graph convolutional neural network framework for assigning partial charges in the future. Initially this effort will focus on reproducing our existing AM1-BCC charge model. We have trained a model using the GraphSAGE inductive framework that generates charges based on a modified version of the charge equilibration scheme proposed by Gilson and co-workers.^158^ In our model we predict an initial partial charge *q*_0,*i*_, the electronegativity *e*_*i*_ and the hardness *s*_*i*_. The model is fitted to a multitarget loss function, considering both actual charges and properties such as dipole moments and the ESP projected by the charges. The model, codenamed NAGL, is orders of magnitude faster than using existing cheminformatic toolkits, e.g. OpenEye or antechamber, to generate AM1-BCC charges for large molecules. A release candidate model is already available for public use with the OpenFF Toolkit from version 0.14.4 onward, and a NAGL model will be used as the canonical charge model for a future release of the Rosemary protein force field.

### 3.4 Virtual Sites

As discussed above, OpenFF force fields currently model electrostatics using a set of AM1-BCC point charges located at the center of atoms. However, atom-centered charges alone cannot accurately capture anisotropies in the electrostatic potential.^159^ The use of additional off-center charges, or virtual sites, can alleviate this issue.^160−164^ OpenFF plans to release a set of force fields, containing virtual sites around moieties that cannot be well-represented using atom-centered charges alone. These virtual sites will be fit to best reproduce the electrostatic potential surface around each molecule. The initial focus will be on halogen σ-holes and lone pairs on pyridine.^165^ In future iterations, we will include additional virtual sites on sulfur and nitrogen groups, as well as investigating using higher and more accurate levels of theory for generating electrostatic potentials.

### 3.5 Water Co-optimization

Fundamentally, simulated properties in aqueous solution are functions not just of the quality of the force fields of the molecules dissolved, but the water model as well. Many water models have been developed that give improved behavior in the bulk phase compared to older water models, but models like TIP3P, with relatively egregious deficiencies in bulk water properties over temperature and pressure, have still been in use in the majority of biopolymer studies. This is due to both some fortuitous cancellations of error in interactions with small molecules and TIP3P,^166^ as well as development of most protein models to have proper behavior in TIP3P water,^33^ though some biomolecular force fields are now working to move beyond this limitation.^167^

Rigorous co-optimization of both water and small molecule parameters has been challenging. However, with the software infrastructure framework provided by OpenFF, such large scale optimization efforts with multiple thermodynamic properties are now feasible. In fact, co-optimization of van der Waals parameters and water has already been carried out in the development and testing of the double exponential potential for van der Waals parameters with demonstrated accuracy benefits;^36^ OpenFF is engaged in further testing and generalization in order to release force fields consistent across solution chemistry and biomolecules with co-optimized water models.

### 3.6 Using Physical Property Surrogate Models to Perform Accelerated Multifidelity Optimization of Force Field Parameters

One of the biggest challenges of using experimental thermodynamic information to optimize force fields is that it requires an enormous number of simulation. Simple thermodynamic observables, such as densities or enthalpies (either of vaporization or mixing^87^) are individually relatively cheap, but a extremely large number of simulations are often needed to perform full parametrizations; first to simulate large numbers of molecules in a single evaluation of a force field, and then to repeatedly evaluate force field performance during the process of a large, multidimensional optimization.

At the present time, we have used OpenFF Evaluator to rapidly perform hundreds of small molecule simulations at a time. This OpenFF framework has allowed optimizations over dozens of Lennard-Jones parameters over hundreds and even thousands of small molecule thermodynamic calculations possible through tens of iterations of regularized least-squares optimization.^39^ However, robust optimization in a rugged force field objective function requires searching through possible parameters some orders of magnitude more efficiently.

One such strategy is that of surrogate modeling, which has been developed for a number of optimization problems,^168−170^ and recently has started to be used for force field optimization.^171,172^ As part of the OpenFF effort, we have tested surrogate modeling to optimize van der Waals parameters to reproduce densities as a function of composition as well as heats of mixing with Gaussian process surrogate modeling.^173^ In particular, we use adaptive multifidelity modeling, where exhaustive searches in Gaussian processes models fit to experiment are interspersed with additional simulations to both refine and broaden the surrogate model of thermophysical properties as a function of force field parameters. Using this technique on two previously studied training sets, containing up to 195 physical property targets, we refit a subset of the LJ parameters for the OpenFF 1.0.0 force field, and found a multifidelity technique can find improved parameter sets compared to a purely simulation-based optimization by searching more broadly and escaping local minima. Additionally, this technique often finds significantly different parameter minima that have comparably accurate performance. In most cases, these parameter sets are transferable to other similar molecules in a test set, with the most transferable force fields being the ones that used the largest and most diverse physical data set for fitting.

Over the next few years, we plan on expanding the capabilities of the main Open Force Field optimization framework to more routinely incorporate multifidelity surrogate modeling approaches, including extensions such as Bayesian optimization. Such extensions will make it much faster to perform more extensive and rigorous force field optimization and answer, more quantitatively, questions about whether a given force field is better than others. For example, surrogate models of thermodynamic properties as a function of force field parameters, if sufficiently accurate, would make it possible to perform Bayesian analysis with the resulting likelihood function and compare force fields in a robust statistical way.

### 3.7 The Design Space between Molecular Mechanics (MM) and Machine Learning Potentials

Recently, machine learning force fields^174−178^ have risen to become a popular alternative to traditional MM force fields. A common technique is to use a (sometimes universal) E(3) or SO(3) equivariant graph neural network^179^ to model the mapping from the joint space of semantic representation and geometry to a scalar-valued energy. Automatic differentiation is usually used to come up with force predictions from energy predictions, ensuring the conservation thereof. Even in low-energy regions, the energy disagreement between MM and QM models usually surpasses 1 kcal/mol—the empirical threshold termed *chemical accuracy* beyond which a model can faithfully reproduce the qualitative behavior of physical systems—whereas that between machine learning potentials and QM is usually less than 0.1 kcal/mol on popular benchmark data sets. On the other hand, machine learning potentials are usually an order of magnitude slower than MM force fields and can suffer from numerical instability due to their sophisticated functional forms;^180^ these functional forms can apparently create high-energy configurations unvisited in training which can cause stability issues. Another source of error may be deficiencies in capturing long-range interactions, for example in potentials that do not incorporate message-passing.

There have been efforts to cross or remove the boundary between MM-based and machine learning potential-based molecular dynamics.^181^ In the next decade, we believe, with the development of modern hardware and middleware, more methods like this will emerge. We are interested in the design space between MM and machine learning models—simple yet flexible functional forms balancing interpretability and flexibility, stability and expressiveness, and speed and accuracy. For instance, one can construct highly expressive (or even universal) functional forms using only dot-product scalarizations on equivariant features^176,182^ without employing spherical harmonics. Alternatively, Class II force fields^183−185^ can be enriched and expanded to incorporate highly flexible terms. We hope that the data and infrastructure developed at Open Force Field will keep contributing to the development of force fields of high utility.

## 4 Conclusions

The OpenFF Initiative is a network of academic and industry researchers working together to advance science and infrastructure required for building the next generation of small molecule and biomolecular force fields and, perhaps more importantly, force field building infrastructure. The shared goal of these efforts is to develop automated and systematic data-driven techniques to parametrize and assess new generations of more accurate force fields. Software and data are released under open licensing agreements to enable rapid application, validation, extension and any kind of modification by users and contributors in the field. In addition, the Initiative aims to build and support a strong community of users and contributors from industry and academia, while exploring different pathways to sustainability.

OpenFF seeks to accelerate force field science by ensuring our data and fitting infrastructure can be reused by diverse researchers across the field, allowing a democratization of force field progress and science. As authors, we hope for a future where diverse researchers from a variety of scientific fields can easily experiment with fitting force fields of a variety of functional forms and domains of applicability, choosing to systematically vary choices such as

1. Type and diversity of fitting data
2. Fitting procedure and fitting targets/objectives
3. Force field functional form or class
4. Balance of experimental vs computational reference data for fitting

and many other aspects, performing fitting experiments to determine exactly how these choices impact force field accuracy and transferrability for selected application domains. This will only be possible to the extent that the field begins to standardize around force field representation and portability, and works to ensure that putative force field tests actually hold the computational method fixed and only vary the choice of force field.

The work of Conflitti, Raniolo and Limongelli^186^ neatly summarizes the need for both openness and standardization in this area as follows: “...we believe existing and new FFs should be developed following the principles of data openness. Most FFs use diverse definitions for residue names, atom names, or types, which may confuse a novice user. In addition, they have different parametrization routines, which often involve diverse pools of model compounds... Developing standardized FFs with improved physicochemical description, uniform parametrization protocols, unified validation tests, and reproducible results over a wide array of functional groups would be advantageous for accurately predicting kinetic data and MD calculations... In this context, abandoning the historical classification of atomic entities into atom types, which unnecessarily complicates present FFs due to redundancy issues, in favor of alternative approaches such as the one presented by [OpenFF] could support the development of a gold standard. A first step in this direction could be the development of public repositories of model compounds with theoretical and experimental data for the parametrization of FFs to avoid discrepancies in the reference data pools. To date, the Open Force Field initiative is the only consortium to host the complete data set employed for the parametrization of its FF in an openly accessible form.”

We indeed hope that the OpenFF Initiative can help further these goals that as a field, we can openly share and collaborate on open force field standards, data sets, and tools in order to accelerate progress in this vital area underlying so much of molecular modeling.
